# Efficacy and safety of antiviral therapy for HBV in different trimesters of pregnancy: systematic review and network meta-analysis

**DOI:** 10.1007/s12072-020-10026-0

**Published:** 2020-03-19

**Authors:** Yuchao Wu, Jinfeng Liu, Yali Feng, Shan Fu, Fanpu Ji, Long Ge, Naijuan Yao, Xufei Luo, Yingren Zhao, Yaolong Chen, Yuan Yang, Tianyan Chen

**Affiliations:** 1 Department of Infection Disease and Hepatopathy, First Affiliated Hospital of Xi’an Jiaotong University, Xi’an, 710061 Shaanxi China; 2 Department of Infection Disease and Hepatopathy, Second Affiliated Hospital of Xi’an Jiaotong University, Xi’an, Shaanxi China; 3 Evidence-Based Medicine Center, Basic Medical Sciences, Lanzhou University, Lanzhou, China; 4 WHO Collaborating Centre for Guideline Implementation and Knowledge Translation, Lanzhou, China

**Keywords:** Hepatitis B, Mother-to-child transmission, Pregnancy, Antiviral treatment, Network meta-analysis, Lamivudine, Telbivudine, Tenofovir, Efficacy, Safety outcomes

## Abstract

**Background:**

Several antiviral agents licenced for blocking mother-to-child transmission (MTCT) of HBV, but their relative efficacy beginning from *different trimesters* has scarce been evaluated. We aimed to conduct a network meta-analysis to statistically differ the efficacy and safety of each antiviral agents initiating on different timings in preventing mother-to-infant transmission of HBV.

**Methods:**

Studies were included from PubMed, EMBASE, Web of Science, and Cochrane databases through July 1, 2019. Eligible studies recruited randomized controlled trials and nonrandomized studies reporting about infant or/and maternal efficacy and safety outcomes and were screened by two investigators independently. Extracted data were analyzed by pair-wised and network meta-analysis, respectively.

**Results:**

3 Randomized and 32 nonrandomized studies enrolling 6738 pregnant female were included. Using network analysis, any antiviral agent interrupted HBV vertical transmission much more effectively than placebo. No agent showed significant efficacy different from others, but a strong trend toward significance was found in telbivudine and tenofovir, of which had the highest probability of being ranked the first- or second-best treatment for reducing MTCT of HBV. The treatment applied in the first and second trimester had a similar efficacy in preventing MTCT. Compared with the initiation during the third trimester, lower rate of MTCT was revealed when antiviral therapy was administrated before third trimester, (RR = 0.045, 95% CI 0.0053 to 0.20); a similar effect at delivery on suppressing maternal HBV DNA level and converting serum HBeAg were achieved if the timing of antiviral treatment started prior, but an obvious improvement of normalizing ALT flare was calculated out; no statistically differences among maternal and fetal safety outcomes were found if mothers received antiviral agents before pregnant 28 weeks.

**Conclusion:**

This network meta-analysis recommended the earlier use of telbivudine or tenofovir, tends to be better to prevent MTCT of HBV in pregnancy with no increased adverse maternal or fetal outcomes.

**Electronic supplementary material:**

The online version of this article (10.1007/s12072-020-10026-0) contains supplementary material, which is available to authorized users.

## Introduction

Perinatal or intrauterine transmission is the major risk of transmission of HBV in its area of high endemicity [1]. Since the last decades, hepatitis B vaccination and immunoglobulin administration provided at birth have been implemented to prevent vertical transmission of HBV [2]. However, 50 Mio. new cases of HBV infection are still diagnosed annually due to mother-to-child transmission, MTCT [3]. High level of HBV DNA level (HBV > 10^6^ IU/mL) and HBeAg positivity during reproductive years [4] contribute to the high risk of transmission, particularly in Asian countries [5].

Lamivudine (LAM), telbivudine (LDT), and tenofovir (TDF) are the oral anti-HBV drugs approved by FDA. All agents have been well investigated and show effective and safe in reducing the risk of HBV MTCT if pregnant female were carrying high viral load of HBV [6]. Emerging data suggest the application of antiviral therapy in the third trimester is able to prevent immunoprophylaxis failure [7–10] to a great extent. Currently, only scarce studies showed the rates of MTCT appeared similar under the utilization of agents during second and third trimester [11], but the events of HBV transmission were still reported even if their mothers obtained a treatment in late pregnancy [12, 13]. The well efficacy and safety in preventing MTCT have been clearly investigated among highly viremic HBV of mothers that started antiviral therapy in the first or second trimesters [12, 14]. Therefore, it’s necessary to identify a more effective and safe period for initiating antiviral treatment (AVT) in pregnancy to completely prevent HBV transmission.

Network meta-analysis is undoubtedly a better way to calculate the risk ratio of rare events. It allows to discriminate the interventions which are lack of head-to-head comparisons [15] by indirect means. Thus, for our primary objective to identify the effective timing of starting AVT, network meta-analysis allowed us to make a distinction of the period between the early-middle pregnancy and late pregnancy on agent use in preventing perinatal transmission. Our secondary objectives were set to determine safety outcomes among different trimesters of treatment administration.

## Materials and methods

See Supplementary Materials**.**

## Results

### Characterization and quality of studies

**Fig. 1:**
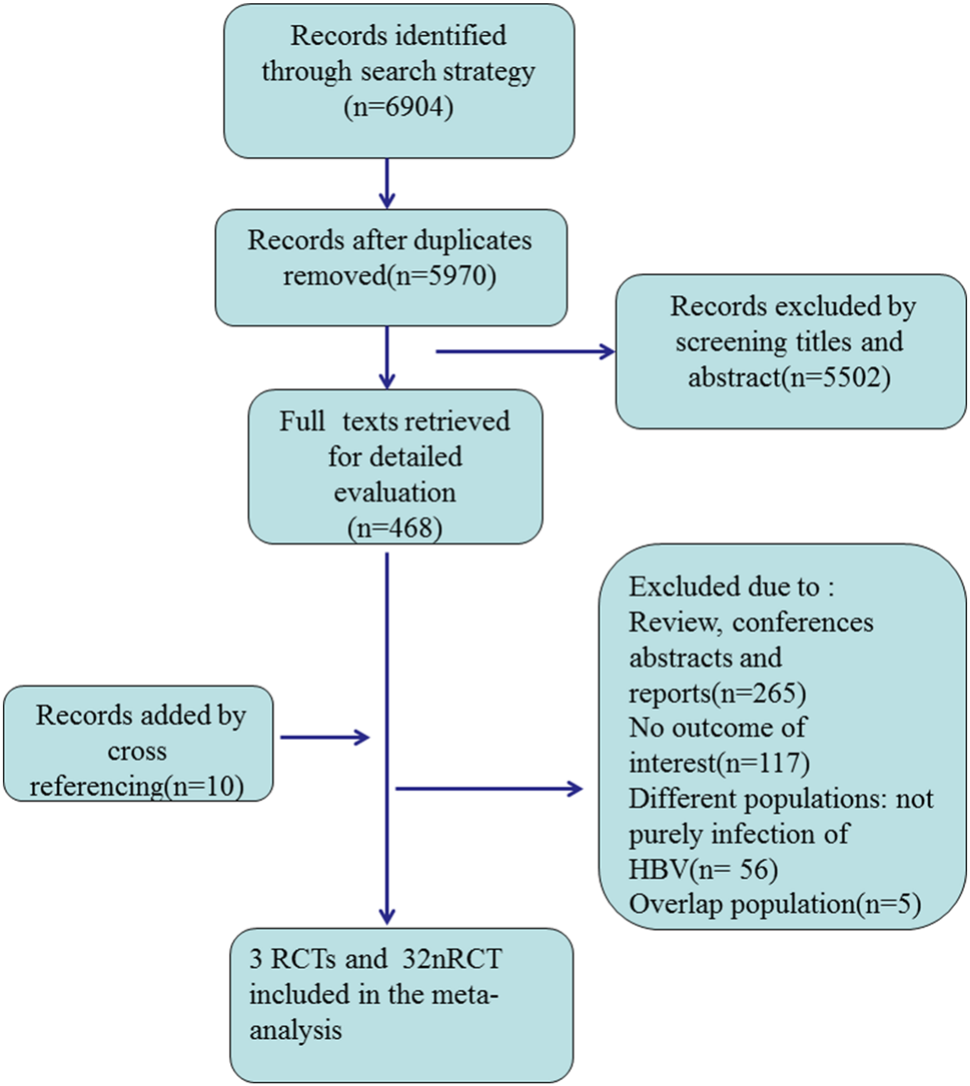
Flow diagram of study selection process in the systematic review

The search strategy generated 6904 citations, 468 of which appeared to be relevant. We retrieved for detailed evaluation after removing records due to duplicated titles and abstracts. Other 10 articles were included by cross referencing. Among those 478 manuscripts, 443 were excluded for various reasons, and finally 35 controlled studies, including 3 RCTs and 32 nonrandomized studies, were selected. 6738 pregnant female were enrolled in our analysis (Fig. 1). 28 in English and 7 in Chinese were fully published. Most of the studies (74%, 26/35) were conducted in China among pregnant female that started AVT across different trimesters because of a high viral load of HBV DNA. All infants received HBV vaccination and immunoglobulin at birth. Table S1 summarized the characterization of individual studies.

Among the selected studies, 3 RCTs [7–9] and 12 non-RCTs [10, 16–26] compared treatment started in the third trimester versus control, 7 non-RCTs compared the timing of second trimester versus control [27–33], 3 non-RCTs compared first trimester versus control [14, 34, 35], 4 non-RCTs compared second and third trimester versus control respectively [11, 36–38], 3 non-RCTs compared first, second trimester versus control respectively [39–41]; 2 non-RCTs compared first, third trimester versus control respectively [12, 42], 1 non-RCTs compared first, second trimester, third trimester versus control respectively [13]. No randomized controlled trials were conducted for the treatment that was applied during first or second trimester except for several non-RCTs.

Risk of biases for all included studies was presented in Tables S2 and S3. 3 RCTs were at low risk of bias. 81% (26/32) non-RCTs were assigned as low level of risk of bias according to the reports of adequate patient selection methods, comparable study groups, and adequate outcome measures as well as follow-up data.

### Efficacy for infant outcomes: pair-wised meta-analysis and network meta-analysis

#### Pair-wised meta-analysis

We performed meta-analysis among pure RCTs and non-RCTs respectively, to obtain their specific grade evidence. Then we incorporated RCTs with non-RCTs data together for a pooled meta-analysis to find out the biased impact of RCTs or non-RCTs on the meta-analysis in the real world. We focused on the comparisons of following timing groups, including gestation ~ 28 weeks and gestation 28–32 weeks, and aimed to get a clear investigation about the optimal initial time point of AVT in reducing MTCT.

**Fig. 2:**
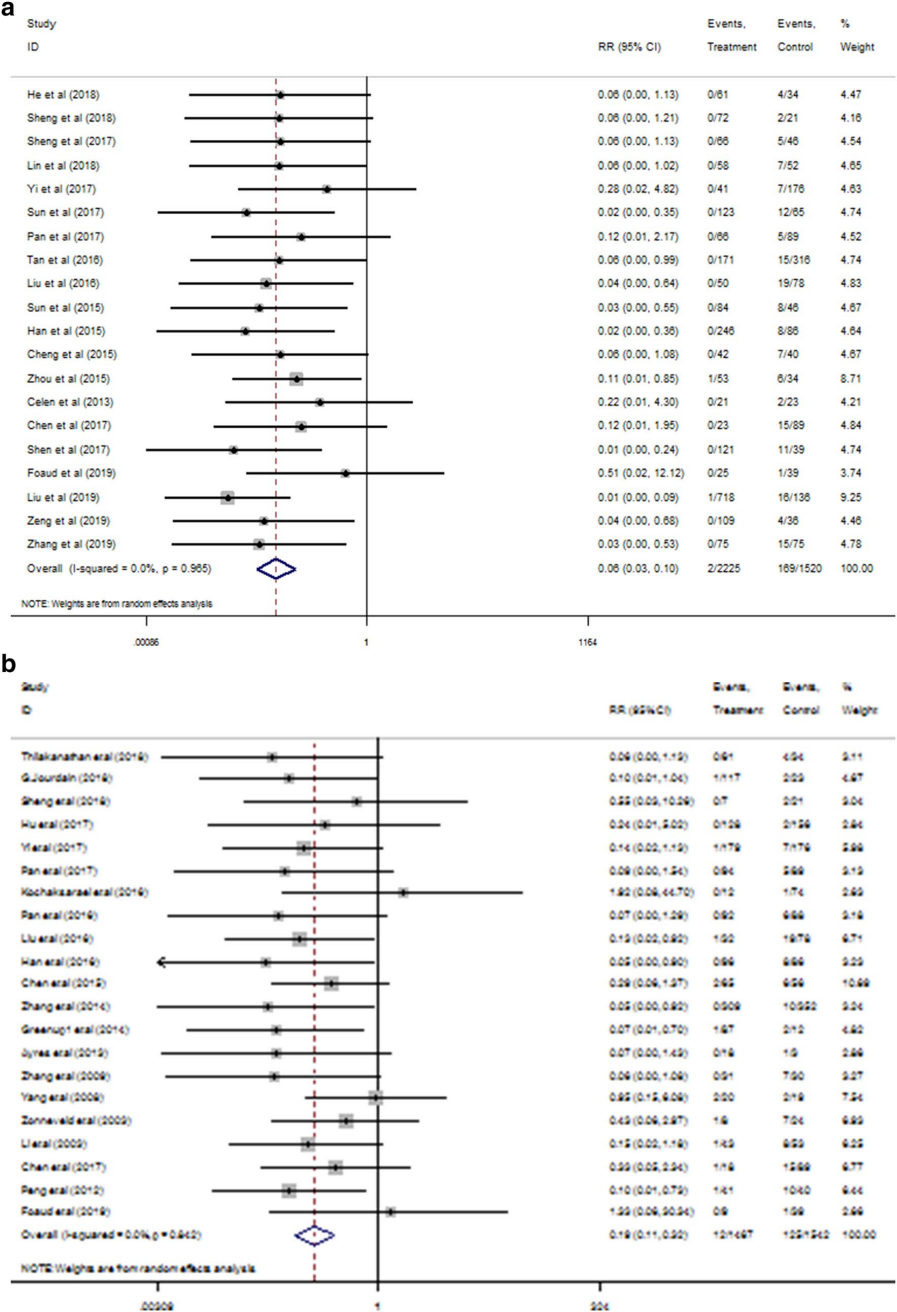

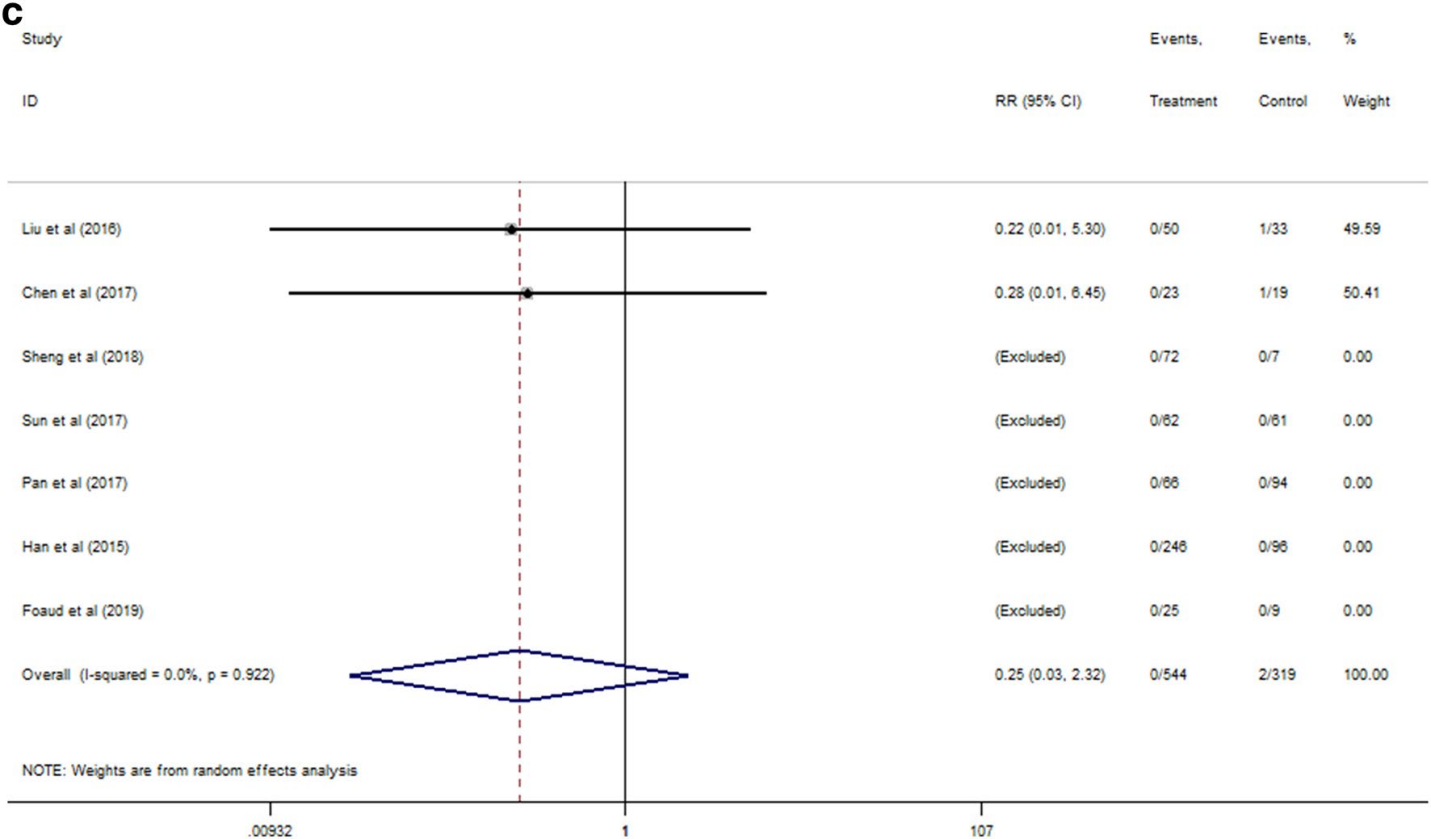
Forest plot of pair-wised meta-analysis of MTCT at 6–12 months after delivery for non-RCTs. **a** Antiviral therapy ~ 28 week versus control, **b** 28–32 week versus control at 6–12 months after delivery. **c** ~ 28 week versus 28–32 week

Comparing with control group, conducting any antiviral drugs in early-middle pregnancy (21 non-RCTs; RR 0.06; 95% CI 0.03 to 0.10, Fig. 2a) and late pregnancy (21 non-RCTs: RR 0.19; 95% CI 0.11 to 0.32, Fig. 2b) were both associated with reduced the likelihood of MTCT, based on calculated ratio of HBsAg serum positivity or/and HBV DNA seropositivity for infants at 6–12 months after delivery. AVT used prior late pregnancy although didn’t have significant difference but tended to have in decreasing the rate of HBV vertical transmission, as compared with the treatment administrated in late pregnancy (RR, 0.25; 95% CI 0.03 to 2.32, Fig. 2c). Utilization of any antiviral agents prior gestation 28 week and during 28–32 week reduced the ratio of infected infants by 10.8% and 7.3%, respectively, compared to control. AVT during earlier trimester appeared to reduce the risk by 3.5% over the administration in late pregnancy. This means that the probability of AVT before the third trimester may be more effective in preventing MTCT. The quality of evidence was low and rated down due to the risk of bias in non-RCTs. When studies were pooled, there was no evidence of publication bias or other small-study effects (Fig. S1a, b), with Egger’s test *p* value > 0.05.

We further performed a subgroup analysis from the timing of usage before the third trimester. As respected, either AVT at ~ 14 weeks or between 14 and 28 weeks showed significant efficacy in interrupting MTCT separately when comparing with untreated pregnant female (Fig. S2a, b).

#### Network meta-analysis

**Fig. 3:**
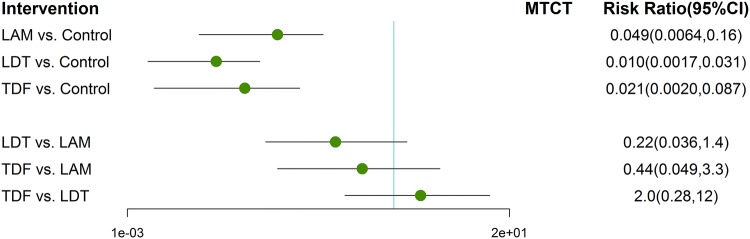
Forest plot for network meta-analysis of MTCT of different agents initiated during pregnancy

**Fig. 4:**
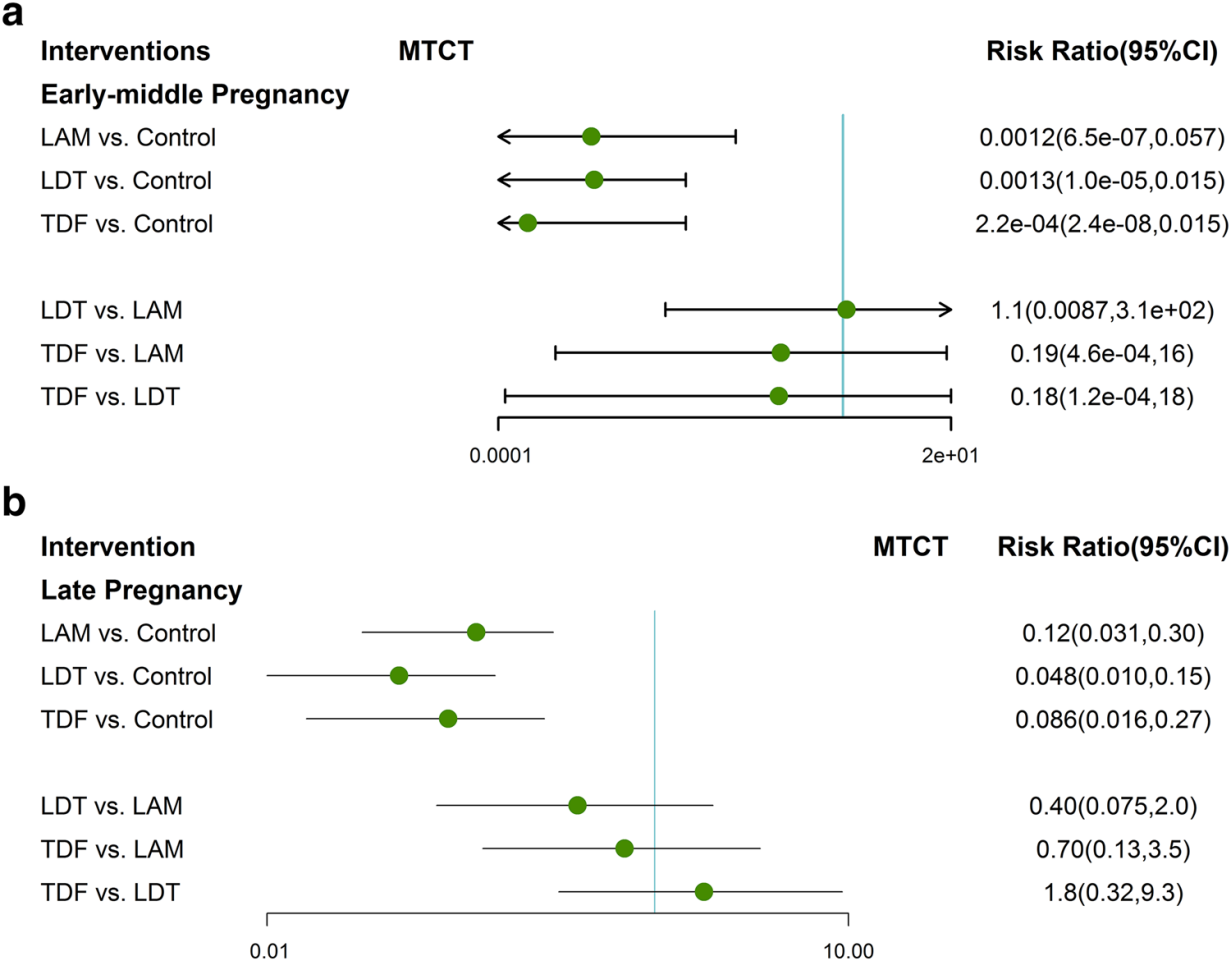
Forest plot for network meta-analysis of MTCT of different agents used **a** before third trimester and **b** in third trimester

Previous studies have indicated LAM, LDT and TDF have great efficacy on decreasing MTCT rate of HBV. At first, we integrated RCTs with non-RCTs to perform an overcalled Bayesian network analysis for the comparisons among the above three agents. To avoid the risk of bias of individual agent effect on the results, we conducted the following meta-analysis to descript the efficacy of different agents initiated in different trimesters on preventing HBV vertical transmission. The pooled network plots were provided in Fig. S3. Compared to untreated group, LAM, LDT and TDF all had significant improvement to reduce HBV transmission (Fig. 3). Though no agent showed significant efficacy different from others (Fig. 3), a strong trend toward significance was found in telbivudine and tenofovir, of which had the highest probability of being ranked the first- or second-best treatment for reducing MTCT of HBV (Figs. S4, S5). In addition, the detailed analysis indicated that the agents initiated either prior gestation 28 week or during 28–32 week were superior than control group on reducing MTCT rates, but no agent was clearly superior to others (Fig. 4).

**Fig. 5:**
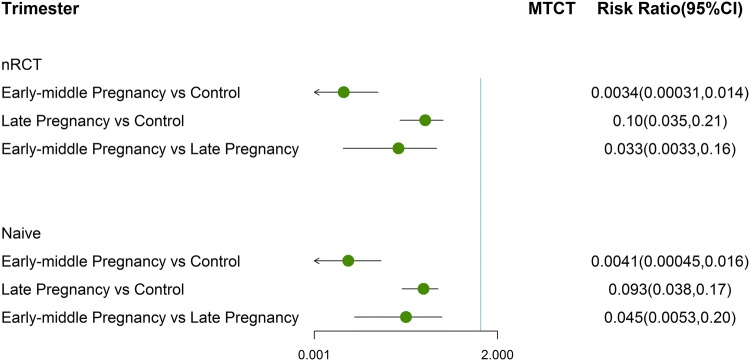
Forest plot for network meta-analysis of MTCT of any agents among different trimesters

Therefore, we continued to conduct the Bayesian network meta-analysis specially non-RCTs to obtain available GRAD evidence. The network and forest plots were provided in Figs. S6 and 5. As compared with the control group, pregnant female that received AVT either prior third trimester (32 non-RCTs: RR 0.0034; 95% CI 3.1E−04 to − 0.014) or in the third trimester (32 non-RCTs: RR 0.10; 95% CI 0.035 to 0.21) got an improvement in preventing MTCT (Fig. 5). The treatment applied in early-middle pregnancy obtained superior efficacy to that intervention starting in late pregnancy (32 non-RCTs: RR 0.033; 95% CI 0.0033 to 0.16, Fig. 5). We further synthesized multiple RCTs and non-RCTs for pooled analysis to evaluate the potential impact of RCTs on the results. The pooled analysis showed similar RR and 95% CI based on the network meta-analysis of pure non-RCTs (Fig. 5).

**Fig. 6:**
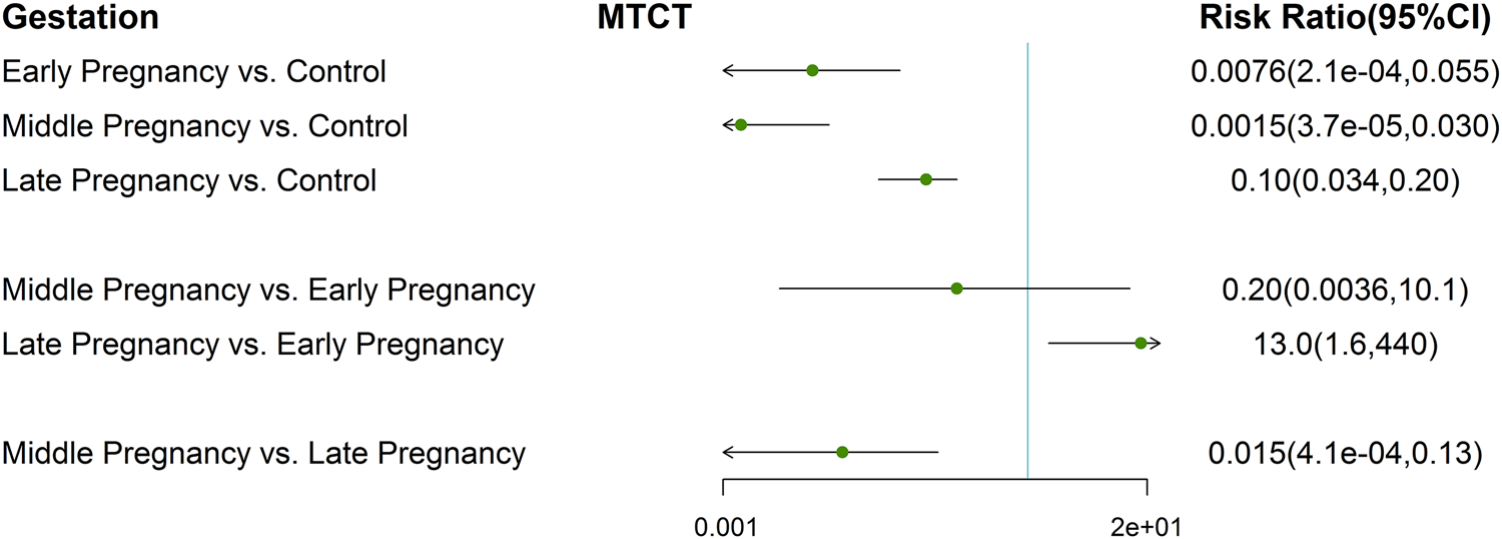
Forest plot for network meta-analysis of MTCT comparing any agents in first, second, third trimester versus control at 6–12 months after delivery in non-RCTs

To reveal whether giving agents in early or middle pregnancy produce a potential impact on the bias of meta-analysis, subgroup analysis demonstrated that timing of treatment in early pregnancy (32 non-RCTs: RR 0.0076; 95% CI 2.1E−04 to 0.055), middle pregnancy (32 non-RCTs: RR 0.0015; 95% CI 3.7E−05 to − 0.03) and late pregnancy (32 non-RCTs: RR 0.10; 95% CI 0.034 to 0.20) all had statistically significant reduction in MTCT at 6–12 months after delivery than untreated mothers. The network and forest plots were provided in FigS. S7 and 6. No significant priority was suggested for the timing selection between second trimester (32 non-RCTs: 0.20; 95% CI 0.0036 to 10.1) and first trimester (Fig. 6) on decreasing HBV MTCT. However, AVT starting during the second trimester attached much more effect on preventing HBV vertical transmission than that in the third trimester (32 non-RCTs: 0.015; 95% CI 4.1E−04 to − 0.13, Fig. 6). Treatments ranking probabilities, from largest to smallest, were as follows: second trimester, first trimester, third trimester, control (Fig. S8).

In addition, we conducted network meta-analysis among the studies enrolling HBeAg positive mothers. Among the HBeAg positive pregnant female received AVT treatment, initiation in middle pregnancy indicated an improved effect on reducing MTCT than initiation in late pregnancy (RR 2.7E−18, 95% CI 6.0E−55, 0.0081, Fig. S9).

### Efficacy for maternal outcomes: network meta-analysis for non-RCTs

We focused on the rate of HBV DNA suppression, HBeAg seroconvension and ALT normalization as the maternal efficacy outcomes. The forest plot for the Bayesian network meta-analysis was provided in Fig. S10. Comparing with the untreated group, intervening in earlier and late pregnancy both showed the significance of HBV DNA suppression, HBeAg seroconvension and ALT normalization. Comparing with the AVT initiated in late pregnancy, starting treatment in early-middle pregnancy had similar significant efficacy in HBV DNA suppressions (16 non-RCTs: RR 1.3; 95% CI 0.36 to 4.4), and HBeAg seroconversion (6 non-RCTs: RR 0.81; 95% CI 0.12 to 6.4). However, applying AVT in early-middle pregnancy suggested a better improvement in ALT normalization (11 non-RCTs: RR, 1.2; 95% CI 1.1 to 1.3) than in late pregnancy.

### Safety for infant outcomes: network meta-analysis for non-RCTs

When comparing with the control group, administrating antiviral therapy prior or during the third trimester did not show clear difference from others on the incidence of congenital malformation rate, prematurity rate, Apgar scores < 8, fetal death, and low birth weight (Fig. S11).

### Safety for maternal outcomes: network meta-analysis for non-RCTs

When compared with control, conducting any antiviral therapy before or during the third trimester indicated no difference from others according to the incidence of postpartum hemorrhage, cesarean section, and elevated creatine kinase, gestational hypertension, membrane prerupture, oligohydramnios and polyhydramnios (Fig. S12).

### Quality of evidence

The quality of the evidence about maternal and infant outcomes (Table S4) was low to very low due to the risk of bias and imprecision of nonrandomized controlled studies. We did not rate down any comparison for publication bias or indirectness.

## Discussion

In this multiple-treatments meta-analysis of RCTs and nRCTs studies for the prevention of HBV viral MTCT, a comprehensive literature search was conducted with no restriction of publication date to ensure maximum coverage of existing studies. Two independent reviewers, Feng and Wu, conducted this research and included conference proceedings. The assessment of eligibility and data extraction were also performed in duplication by two independent reviewers. We subsequently contacted the authors of 5 other studies in which timing of agent application crossed gestation 28 weeks [7, 37, 38, 43, 44], to obtain necessary data unable to get in original papers. However, we were not able to obtain required data from 3 studies that AVT was given on the timing of 24–32 and 24–36 week [7, 43, 44]. We used a per-protocol analysis with a random-effects model to minimize the risk of MTCT that would be overestimated. Finally, we extracted and pooled maternal and infant outcomes, if reported, to provide a summary of safety data.

Major clinical guidelines recommended the third trimester as the inception date due to inadequate evidence of safety in human data evaluated in an earlier pregnancy. Previous study has published that LDT administrated throughout early, middle, or late pregnancy is safe and effective to interrupt mother-to-infant transmission [39]. Therefore, it needs to define optimal timing to initiate antiviral prophylaxis to completely prevent MTCT of HBV during pregnancy [45].

This network meta-analysis confirmed that administration of antiviral agents in early-middle pregnancy was associated with lower MTCT of HBV than the use in late pregnancy. LDT and TDF had a trend of prior use on reducing MTCT of HBV. Furthermore, starting treatment prior gestation 28 weeks was associated with enhancement of maternal ALT normalization at delivery. No significant differences in safety outcomes for both mothers and infants were found according to comparison with the treatment provided in the third trimester and control group.

High viral load is independently associated with the high risk of HBV MTCT. For mothers at high risk, initiating MTCT in middle pregnancy, at least, can obtain adequate time to suppress the level of HBV DNA. In addition, the earlier administration is capable to control mothers’ liver function and decrease the risk of viral breakthrough during pregnancy. Current clinical guidelines reveal a range of HBV cutoff to prevent HBV transmission from 2 × 10^5^ to 2 × 10^7^ IU/mL [46, 47]. Amongst major studies, pregnant mothers were enrolled due to the high level of HBV DNA level (HBV DNA > 6 log IU/mL). The average HBV DNA level was 7.074 (6.986, 7.161) log_10_ IU/mL calculated by a random effect model (base characteristics of HBV DNA found in the Supplementary Material). However, we were unable to evaluate the effect on decreasing MTCT among the maternal HBV DNA level below 2 × 10^5^ IU/mL due to the lack of researches. In addition, among the HBeAg positive pregnant female received AVT treatment, initiation in middle pregnancy indicated an improved effect on reducing MTCT than initiation in late pregnancy. Accordingly, antiviral therapy should be at least provided to HBeAg positive pregnant mothers with high viral loads (> 2 × 10^6^ IU/mL).

Several systematic reviews and meta-analysis have been performed to evaluate the efficacy and safety of all the FDA-recommended antiviral agents including LAM, LDT and TDF in interrupting MTCT of HBV. Brown et al. recommended the use of these agents in female who are HBeAg positive and high level of HBV DNA in the third trimester to prevent MTCT [6]. Another meta-analysis showed all recommended agents have a great effect on preventing vertical transmission of HBV and second trimester was the better timing of initiating AVT in the subgroup analysis of HBV DNA/HBsAg positivity [48]. Major of them were based on the pair-wised meta-analysis [6, 48]. Therefore, different from existing studies, our network analysis has some following remarkable characteristics. Firstly, conventional meta-analysis usually focused on pair-wised comparisons of two therapeutic measures, most of which contained controlled and untreated groups. The network meta-analysis was able to construct two or more interventions into a network structure, enabling overall computation of relative effectiveness from both direct one-to-one and indirect comparison with multiple interventions. Second, previous meta-analyses used the frequency method, which might reduce the accuracy of RR, to estimate relative risk for the rare events of HBV MTCT. Bayesian method and ranked probabilities were suitable to provide more precise RR for the rare event of MTCT. However, our analysis has some limitations. First, all the three RCTs were designed for pregnant patients during the third trimester, and none of RCTs were conducted for patients before the third trimester. No head-to-head RCTs identified the efficacy of antiviral drugs started among different trimesters that our analysis mainly coming from non-RCTs cannot comprehensively reflect antiviral efficacy and lower down the grade evidence of observation in the real-world. As most of the guidelines recommend starting AVT in the third trimester, so it is unethical to perform RCTs to compare early treatment during pregnancy with no treatment group in future. In addition, due to the rare event of HBV MTCT after antiviral therapy during pregnancy, conducting a randomized trial comparing antiviral therapy initiated in the second vs. third trimester will be logistically difficult (huge sample size required). Network meta-analysis could collect direct and indirect evidence of non-RCTs to reduce statistical power and uncertainty on ranking results, and be effectively closed to the facts in the real world. Second, because most of non-RCTs provided different intervals of timing for treatments, the quality of data on this crucial question was low because exact initial timing of antiviral therapy was not specified or the timing was provided as a wide range in many studies. Third, the distributions of patients and disease background exist with selective bias based on non-randomized studies. In addition, some studies were conducted many years ago that the definition of MTCT of HBV was mainly based on HBsAg seropositivity at 6 months after delivery and subdata (e.g. maternal and infant outcomes) were not completely collected. We cannot draw a statistically meaningful conclusion when we analyze those safety data due to the limitation of information.

In summary, this network meta-analysis suggests AVT administered in each trimester from first to the second trimester appeared superior to prevent MTCT of HBV than the third trimester, for HBeAg positive pregnant mothers with high viral loads (> 2 × 10^6^ IU/mL). The limited safety data regarded to maternal and infant outcomes demonstrated no significant increased risk of issues. Large comparative RCTs, particularly of the utilization of AVT in early pregnancy or/and the head-to-head trials are warranted to further establish optimum initial timing to completely block HBV MTCT.

## Electronic supplementary material

Below is the link to the electronic supplementary material.

Supplementary file1 (DOCX 2573 kb)

## References

[1] Schweitzer A, Horn J, Mikolajczyk RT, Krause G, Ott JJ (2015). Estimations of worldwide prevalence of chronic hepatitis B virus infection: a systematic review of data published between 1965 and 2013. Lancet (Lond Engl).

[2] Kane MA (1996). Global status of hepatitis B immunisation. Lancet (Lond Engl).

[3] Buchanan C, Tran TT (2010). Management of chronic hepatitis B in pregnancy. Clin Liver Dis.

[4] Lok AS, Lai CL, Wu PC, Leung EK, Lam TS (1987). Spontaneous hepatitis B e antigen to antibody seroconversion and reversion in Chinese patients with chronic hepatitis B virus infection. Gastroenterology.

[5] Livingston SE, Simonetti JP, Bulkow LR (2007). Clearance of hepatitis B e antigen in patients with chronic hepatitis B and genotypes A, B, C, D, and F. Gastroenterology.

[6] Brown RS, McMahon BJ, Lok AS (2016). Antiviral therapy in chronic hepatitis B viral infection during pregnancy: a systematic review and meta-analysis. Hepatology.

[7] Xu WM, Cui YT, Wang L (2009). Lamivudine in late pregnancy to prevent perinatal transmission of hepatitis B virus infection: a multicentre, randomized, double-blind, placebo-controlled study. J Viral Hepat.

[8] Pan CQ, Duan Z, Dai E (2016). Tenofovir to prevent hepatitis B transmission in mothers with high viral load. N Engl J Med.

[9] Jourdain G, Ngo-Giang-Huong N (2018). Tenofovir versus placebo to prevent perinatal transmission of hepatitis B. N Engl J Med.

[10] Zhang H, Pan CQ, Pang Q, Tian R, Yan M, Liu X (2014). Telbivudine or lamivudine use in late pregnancy safely reduces perinatal transmission of hepatitis B virus in real-life practice. Hepatology.

[11] Pan CQ, Yi W, Liu M, Wan G, Hu YH, Zhou MF (2017). Lamivudine therapy during the second vs the third trimester for preventing transmission of chronic hepatitis B. J Viral Hepat.

[12] Yi W, Li MH, Xie Y (2017). Prospective cohort study on the efficacy and safety of telbivudine used throughout pregnancy in blocking mother-to-child transmission of hepatitis B virus. J Viral Hepat.

[13] Liu Y, Wang M, Yao S (2016). Efficacy and safety of telbivudine in different trimesters of pregnancy with high viremia for interrupting perinatal transmission of hepatitis B virus. Hepatol Res Off J Jpn Soc Hepatol.

[14] He T, Bai Y, Cai H (2018). Safety and efficacy of lamivudine or telbivudine started in early pregnancy for mothers with active chronic hepatitis B. Hepatol Int.

[15] Cipriani A, Higgins JP, Geddes JR, Salanti G (2013). Conceptual and technical challenges in network meta-analysis. Ann Intern Med.

[16] Thilakanathan C, Wark G, Maley M (2018). Mother-to-child transmission of hepatitis B: examining viral cut-offs, maternal HBsAg serology and infant testing. Liver Int Off J Int Assoc Study Liver.

[17] Hu Y, Xu C, Xu B (2018). Safety and efficacy of telbivudine in late pregnancy to prevent mother-to-child transmission of hepatitis B virus: a multicenter prospective cohort study. J Viral Hepat.

[18] Samadi Kochaksaraei G, Castillo E, Osman M (2016). Clinical course of 161 untreated and tenofovir-treated chronic hepatitis B pregnant patients in a low hepatitis B virus endemic region. J Viral Hepat.

[19] Chen HL, Lee CN, Chang CH (2015). Efficacy of maternal tenofovir disoproxil fumarate in interrupting mother-to-infant transmission of hepatitis B virus. Hepatology.

[20] Greenup AJ, Tan PK, Nguyen V (2014). Efficacy and safety of tenofovir disoproxil fumarate in pregnancy to prevent perinatal transmission of hepatitis B virus. J Hepatol.

[21] Ayres A, Yuen L, Jackson KM (2014). Short duration of lamivudine for the prevention of hepatitis B virus transmission in pregnancy: lack of potency and selection of resistance mutations. J Viral Hepat.

[22] Zhang LJ, Wang L (2009). Blocking intrauterine infection by telbivudine in pregnant chronic hepatitis B patients. Chin J Hepatol.

[23] Yang S, Liu M, Wang L (2008). Effect of high viral hepatitis B virus DNA loads on vertical transmission of hepatitis B virus in late-pregnant women. Zhonghua fu chan ke za zhi.

[24] van Zonneveld M, van Nunen AB, Niesters HG, de Man RA, Schalm SW, Janssen HL (2003). Lamivudine treatment during pregnancy to prevent perinatal transmission of hepatitis B virus infection. J Viral Hepat.

[25] Li XM, Yang YB, Hou HY (2003). Interruption of HBV intrauterine transmission: a clinical study. World J Gastroenterol.

[26] Peng BA, Zhao Y, Yang XF, Miao MF, Zhu LH, Yu HY (2012). Evaluation of the efficacy and safety of telbivudine in preventing mother-to-infant HBV transmission. Chin Pharm J.

[27] Sheng QJ, Wang SJ, Wu YY, Dou XG, Ding Y (2018). Hepatitis B virus serosurvey and awareness of mother-to-child transmission among pregnant women in Shenyang, China: an observational study. Medicine.

[28] Lin Y, Liu Y, Ding G (2018). Efficacy of tenofovir in preventing perinatal transmission of HBV infection in pregnant women with high viral loads. Sci Rep.

[29] Celen MK, Mert D, Ay M (2013). Efficacy and safety of tenofovir disoproxil fumarate in pregnancy for the prevention of vertical transmission of HBV infection. World J Gastroenterol.

[30] Shen ML, Xu HT, Ju HF, Xian JC, Yang XZ (2016). Sequential telbivudine/lamivudine and hepatitis B immunoglobulin therapy for preventing mother-to-infant transmission of hepatitis B virus. World Chin J Digestol.

[31] Liu J, Wang J, Yan T (2019). Efficacy and safety of telbivudine and tenofovir disoproxil fumarate in preventing hepatitis B vertical transmission: a real-life practice. J Viral Hepat.

[32] Zeng J, Zheng C, Li H (2019). Effectiveness of tenofovir or telbivudine in preventing HBV vertical transmission for pregnancy. Medicine.

[33] Zhang BF, Cheng ML, Zhang Q (2018). Clinical study on blocking mother-to-child transmission of hepatitis B virus with high viral load and HBeAg positivity during pregnancy in Guizhou Province. Chin J Hepatol.

[34] Chen C, Tu X, Cheng Q (2015). Clinical observation of telbivudine’s antiviral efficacy and protection against mother-to-infant transmission of chronic hepatitis B during the first trimester of pregnancy. Chin J Hepatol.

[35] Zhou Y, Zheng J, Pan H, Lu C (2014). Long-term efficacy and safety of telbivudine in the treatment of childbearing patients with chronic hepatitis B. Chin J Hepatol.

[36] Han GR, Jiang HX, Yue X (2015). Efficacy and safety of telbivudine treatment: an open-label, prospective study in pregnant women for the prevention of perinatal transmission of hepatitis B virus infection. J Viral Hepat.

[37] Chen ZX, Gu GF, Bian ZL (2017). Clinical course and perinatal transmission of chronic hepatitis B during pregnancy: a real-world prospective cohort study. J Infect.

[38] Sheng Q, Ding Y, Li B (2018). Efficacy and safety of nucleos(t)ide analogues to prevent hepatitis B virus mother-to-child transmission in pregnant women with high viremia: real life practice from China. Int J Med Sci.

[39] Sun W, Zhao S, Ma L (2017). Telbivudine treatment started in early and middle pregnancy completely blocks HBV vertical transmission. BMC Gastroenterol.

[40] Tan Z, Yin Y, Zhou J, Wu L, Xu C, Hou H (2016). Telbivudine treatment of hepatitis B virus-infected pregnant women at different gestational stages for the prevention of mother-to-child transmission: outcomes of telbivudine treatment during pregnancy. Medicine.

[41] Sun W, Ma L, Hao A (2015). Predictive value of telbivudine in preventing mother-to-infant transmission of hepatitis B virus in pregnant women with high viremia. Chin J Hepatol.

[42] Foaud HM, Maklad S, Gmal El Din A, Mahmoud F (2019). Lamivudine use in pregnant HBsAg-females effectively reduces maternal viremia. Arab J Gastroenterol Off Publ Pan-Arab Assoc Gastroenterol.

[43] Deng Y, Wu W, Zhang D (2015). The safety of telbivudine in preventing mother-to-infant transmission of hepatitis B virus in pregnant women after discontinuation. Chin J Hepatol.

[44] Wu Q, Huang H, Sun X (2015). Telbivudine prevents vertical transmission of hepatitis B virus from women with high viral loads: a prospective long-term study. Clin Gastroenterol Hepatol Off Clin Pract J Am Gastroenterol Assoc.

[45] Pan CQ, Lee HM (2013). Antiviral therapy for chronic hepatitis B in pregnancy. Semin Liver Dis.

[46] Hou J, Cui F, Ding Y (2019). Management algorithm for interrupting mother-to-child transmission of hepatitis B virus. Clin Gastroenterol Hepatol Off Clin Pract J Am Gastroenterol Assoc.

[47] Chen HL, Wen WH, Chang MH (2017). Management of pregnant women and children: focusing on preventing mother-to-infant transmission. J Infect Dis.

[48] Song J, Yang F, Wang S (2019). Efficacy and safety of antiviral treatment on blocking the mother-to-child transmission of hepatitis B virus: a meta-analysis. J Viral Hepat.

