# Supplementary material for: Efficacy and safety of antiviral therapy for HBV in different trimesters of pregnancy: systematic review and network meta-analysis
Source: Hepatol Int. 2020 Mar 19;14(2):180–9. doi: 10.1007/s12072-020-10026-0 (PMC7136311; doi:10.1007/s12072-020-10026-0)
Supplement: Supplementary file 1 — Supplementary file1 (DOCX 2573 kb) [file 12072_2020_10026_MOESM1_ESM.docx]

**SUPPLEMENTARY MATERIALS**

**Materials Methods**

The study protocol was registered in the International Prospective Register of Systematic Reviews (CRD42019128178). This systematic review of literature is performed according to the Preferred Reporting Items for Systematic Reviews and Meta-Analysis Statement and was conducted following an *a priori* established protocol and is followed good research practices as outlined in the International Society for Pharmacoeconomics and Outcomes Research report on interpreting indirect treatment comparisons and network meta-analysis for health care decision making[[1](#_ENREF_1)]. We conducted this systemic review for a clinical practice guideline in prevent mother-to-child transmission of HBV in China

**Selection Criteria**

We included controlled or comparative studies in this meta-analysis that met the following criteria: (1) patients- pregnant women purely with chronic HBV infection; (2) intervention – received antiviral therapy(AVT) at different gestation week; (3) comparator –controlled pregnant patients with no treatment or placebo; (4) primary outcomes: prevention of MTCT of HBV; secondary outcomes: clinical efficacy and adverse event in both mothers and newborn/infants. Both English and Chinese studies were enrolled in.

We excluded studies that (1) enrolled infants didn’t received immunization postpartum; (2) pregnant mothers were co-infected with hepatitis C, D, or E virus, human immunodeficiency virus(HIV); (3) evidence of hepatocellular carcinoma or liver decompensation; (4) published as uncontrolled studies, conferences, review, abstract, case report or cost analysis.

**Literature Search Strategy and Study Selection**

Two independent investigators systematically searched multiple electronic databases MEDLINE, EMBASE, Cochrane database and Web of Science from inception through July 1, 2019. We performed the search with following terms: “hepatitis B”, “HBV”, “Hep B”, “pregnancy”, “gravidity”, “mother-to-child transmission”, “mother-to-infant transmission”, vertical transmission, intrauterine transmission”, “newborn”, infant, “fetal”, “foetal”, “utero transmission”, “antiviral treatment”, “antiviral therapy”, “antiviral agents”, “lamivudine”, “telbivudine”, “tenofovir” and “nucleoside analogues”.

Then, two independent reviewers screened titles and abstracts in English or Chinese for potential eligibility in duplicate using an online reference management system. The remaining abstracts were reviewed in full text following the same procedure according to inclusion and exclusion criteria. In addition, we reviewed the references in relevant articles as well as published systematic reviews on this topic to identified any additional literatures which were not included in our search. If multiple publications researched on the same or overlapped population, the most recent or comprehensive data were selected. When detailed results were not available, the corresponding authors were contacted. Any disagreements were reconciled by consensus or by a third reviewer.

**Data Collection and Quality Assessment**

The same two investigators independently performed data extraction and risk of bias assessment. Retrieved data from full text papers included: first author, ethnicity, study design, inclusion criteria, number of participants, age, HBV DNA suppression, alanine aminotransferase (ALT) normalisation, HBeAg seroconversion, hepatitis B surface antigen (HBsAg) positivity within 24 hours after birth and at 6-12 months of age, infant HBV DNA positivity within 24 hours after birth and at 6-12 months of age, and maternal and fetal adverse outcomes. The Cochrane Collaboration’s tool was used to assess the risk of bias in the RCT[[2](#_ENREF_2)]. The risk of bias of observational studies were measured on the basis of the Newcastle-Ottawa scale [[3](#_ENREF_3)]. The assessment of outcome in Newcastle-Ottawa scale indicated measurement of infants’ HBV infection status at least 6 months after the delivery. Discrepancies were resolved by consensus.

**Outcomes Assessed**

The maternal efficacy outcomes included HBV DNA suppression, ALT normalization, and HBeAg seroconversion. MTCT rates were defined as HBsAg or HBV DNA positivity of newborns and of infants at 6-12 months of age. Maternal safety outcomes included creatinine kinase (CK) elevation, serum creatinine (Cr) elevation, ALT flare, caesarean section (CS) and post-partum haemorrhage rate. Fetal safety outcomes included congenital malformation, pre-term birth rate, low birth weight (<2500 g), and fetal death.

**Statistical Analysis**

We used outcome data from per-protocol analysis in individually full text papers to perform this statistical meta-analysis. For the primary outcome, we conducted pairwise and network meta-analysis, respectively.

We utilized the STATA software (version 13.0) for the directed meta-analysis. A random-effect model was performed to estimate pooled relative risk(RR) and 95% confidence interval(CI) for the differences of dichotomous outcomes between two groups[[4](#_ENREF_4)]. Statistical heterogeneity was assessed by using Cochran Q statistic and I^2^ statistic, with a P-value <0.1 and an I^2^ value ≥50% indicating significant heterogeneity. I^2^ value < 50% represented acceptable heterogeneity among the studies[[5](#_ENREF_5)]. Publication bias was calculated by examining funnel plot and assessed by Egger’s regression test[[6](#_ENREF_6)].

To integrate indirect comparisons, we performed the network meta-analysis within a random-effects model of Bayesian framework using Markov chain Monte Carlo methods in JAGS (version 4.3.0), R software (version 3.5.3), rjags and gemtc packages. We conducted the model to compare the efficacy of any two treatments with their function relative to the controlled treatment (i.e., no intervention). Network “consistency” was evaluated by comparing the direct estimates to the indirect estimates, testing whether treatment effects between direct and indirect calculates were the same across all trial[[7](#_ENREF_7)].

We updated the Markov chain Monte Carlo model with 100,000 simulated draws after a burn-in of 6,000 iterations. The median of the posterior distribution based on 100,000 simulations was reported as the point estimate (RR), and the corresponding 95% credible intervals (CrIs, or Bayesian CI) was obtained using the 2.5th and 97.5th percentiles of the posterior distribution, after adjusting for multiple arm trials (model available upon request).

**Quality of Evidence**

The quality of evidence of estimates derived from network meta-analysis was evaluated using the Grading of Recommendations Assessment, Development, and Evaluation (GRADE) approach[[8](#_ENREF_8)]. In this approach, we separated analysis of evidence in RCTs and nonrandomized studies (non-RCTs). The direct evidence from non-RCTs starts at low quality and can be rated up or down, based on risk of bias, indirectness, imprecision, inconsistency (or heterogeneity), and/or publication bias, to levels of high, moderate, low, and very low quality. The rating of indirect estimates starts at the lowest rating of the two pair-wise estimates that contribute as first-order loops to the indirect estimate, but can be rated down further for imprecision or intransitivity (dissimilarity between studies in terms of clinical or methodological characteristics). If direct and indirect estimates are similar (i.e., coherent), then the higher of their rating can be assigned to the network meta-analysis of estimates. When the direct evidence had higher quality, we used that over the network evidence.

**Table S1 Summary of the characteristics of included clinical trials.**

|  | First author, year | Region  (Ethnicity) | Study Design | Intervention | Participants of mothers(N) | Age(Year) | Baseline HBV DNA level | Baseline ALT level IU/ml | Treatment Started | Baby Sample Size  (followed) | No. of Transm-  issions | Interven-  Tions on  Infants |
| --- | --- | --- | --- | --- | --- | --- | --- | --- | --- | --- | --- | --- |
| 1 | He, et al, 2018 | China  (Asians) | Retrospective cohort study | Lamivudine | 27 | 29.19 ± 2.93 | 7.01 ± 0.91 log10 copies/ml | 188.60 ± 150.31 | First trimester | 29 | 0 | HBIG+  Vaccinatio |
|  |  |  |  | Telbivudine | 32 | 29.18 ± 2.89 | 7.32 ± 0.73 log10 copies/ml | 207.41 ± 142.42 | First trimester | 32 | 0 | HBIG+  Vaccinatio |
|  |  |  |  | Control group | 35 | 28.97 ± 3.58 | 6.95 ± 0.69 log10 copies/ml | 200.95 ± 190.17 | Control group | 34 | 4 | HBIG+  Vaccinatio |
| 2 | Cynthuja Thilakanathan, et al, 2018 | Australia  (NA) | Retrospective cohort study | Lamivudine  And then tenofovir | 117 | NA | >6 log10 IU/mL | NA | Gestation 32 weeks | 117 | 1 | HBIG+  Vaccinatio |
|  |  |  |  | Control group | 23 | NA | >6 log10 IU/mL | NA | Control group | 23 | 2 | HBIG+  Vaccinatio |
| 3 | G.Jourdain, et al(2018) | Thailand  (Asians) | RCT | Tenofovir | 168 | 25.5 (22.6–29.1) | 7.6±1.5 (log10IU/mL) | NR | Geatation 28 weeks | 147 | 0 | HBIG+  Vaccinatio |
|  |  |  |  | Control group | 163 | 26.7 (23.5–30.5) | 7.3±1.7 (log10IU/mL) | NR | Control group | 147 | 3 | HBIG+  Vaccinatio |
| 4 | Sheng, et al(2018) | China  (Asians) | Observational Study | Telbivudine | 66 | 31.3±4.4 | 8.1±0.4 (log10IU/mL) | 27.4±11.0 | Gestation 24 to 28 weeks | 66 | 0 | HBIG+  Vaccination |
|  |  |  |  | Control group | 46 | 30.4±4.2 | 7.9±0.5 (log10IU/mL) | 25.1±12.3 | Control group | 46 | 5 | HBIG+  Vaccinatio |
| 5 | Sheng, et al (2018) | China  (Asians) | Prospective, open-label, multicenter study | Telbivudine | 91 | 27.8±4.17 | 8.15± 0.82 (log10IU/mL) | 26.53±8.32 | Gestation 24 to 32 weeks | 79 | 0 | HBIG+  Vaccinatio |
|  |  |  |  | Control group | 21 | 26.8±3.66 | 8.09 ± 1.04 (log10IU/mL) | 23.62±6.51 | Control group | 21 | 2 | HBIG+  Vaccinatio |
| 6 | Lin, et al  (2018) | China  (Asians) | Multicenter cohort study,  Double - blind study | Tenofovir | 59 | 28.31 ± 3.56 | >6.3 log10IU/mL | 54.62 ± 105.7 | Gestation 24 weeks | 58 | 0 | HBIG+  Vaccinatio |
|  |  |  |  | Control  group | 60 | 28.06 ± 3.42 | >6.3 log10IU/mL | 57.5 ± 103.3 | Control group | 52 | 7 | HBIG+  Vaccinatio |
| 7 | Hu, et al,  (2017) | China  (Asians) | Prospective cohort study | Telbivudine | 149 | 25.9 ± 3.7 | 7.43 ± 1.26 (log10IU/mL) | NR | Gestation 28 to 32 weeks | 128 | 0 | HBIG+  Vaccinatio |
|  |  |  |  | Control group | 179 | 26.4 ± 3.4 | 7.37 ± 1.49 (log10IU/mL) | NR | Control group | 156 | 2 | HBIG+  Vaccinatio |
| 8 | Yi, et al,  (2017) | China  (Asians) | Prospective cohort study | Telbivudine | 41 | 31.54 ± 4.21 | 1.50 ± 0.62 (log10IU/mL) | 15.19 ± 8.53 | Entire pregnancy | 41 | 0 | HBIG+  Vaccinatio |
|  |  |  |  | Telbivudine | 179 | 27.77 ± 3.48 | 8.05 ± 0.37 (log10IU/mL) | 21.58 ± 13.15 | Gestation 28weeks | 179 | 1 | HBIG+  Vaccinatio |
|  |  |  |  | Control group | 176 | 28.27 ± 3.65 | 7.94 ± 0.62 (log10IU/mL) | 18.85 ± 9.83 | Control group | 176 | 7 | HBIG+  Vaccinatio |
| 9 | Sun, et al  (2017) | China  (Asians) | Prospective cohort study | Telbivudine | 62 | 28.9 ± 11.8 | 7.79 ± 0.22 (log10IU/mL) | 125.3 ± 57.6 | Gestation 12 weeks | 62 | 0 | HBIG+  Vaccinatio |
|  |  |  |  | Telbivudine | 61 | 29.7 ± 9.8 | 7.75 ± 0.19 (log10IU/mL) | 132.3 ± 52.9 | Gestation 20 to 28 weesk | 61 | 0 | HBIG+  Vaccinatio |
|  |  |  |  | Control group | 65 | 27.5 ± 12.9 | 7.74 ± 0.22 (log10IU/mL) | 128.5 ± 48.7 | Control group | 65 | 12 | HBIG+  Vaccinatio |
| 10 | Pan, et al  (2016) | China  (Asians) | Retrospective cohort study | Lamivudine | 66 | 27.65 ±4.08 | 7.22±0.61 6 log10Copies/mL | 68.6±103.6 | Second trimester | 66 | 0 | HBIG+  Vaccinatio |
|  |  |  |  | Lamivudine | 94 | 27.37 ±3.54 | 7.26±0.55 log10Copies/mL | 36.4±39.7 | Third trimester | 94 | 0 | HBIG+  Vaccinatio |
|  |  |  |  | Control group | 89 | 27.08 ±4.22 | 7.33±0.47 log10Copies/mL | 28.0±35.4 | Control group | 89 | 5 | HBIG+  Vaccinatio |
| 11 | Tan, et al  (2016) | China (Asians) | Prospective study | Telbivudine | 34 | 29 (23–39) | 2 (1.82–6.99) (log10IU/mL) | 18 (9–500) | Before gestation week 14 | 34 | 0 | HBIG+  Vaccinatio |
|  |  |  |  | Telbivudine | 135 | 29 (20–38) | 7.69 (6.05-8.98) (log10IU/mL) | 37 (6–697) | Gestation week 14 to 28 | 137 | 0 | HBIG+  Vaccinatio |
|  |  |  |  | Control group | 316 | 28 (20–41) | 7.67 (6–8.91) (log10IU/mL) | 22 (5–623) | Control group | 320 | 15 | HBIG+  Vaccinatio |
| 12 | G. Samadi Kocha-ksaraei, et al(2016) | Canada (Asia, Africa, Other) | Prospectively recruited | Tenofovir | 23 | 30 (28–34) | 7.7 (3.2–8.1) (log10IU/mL) | 30 (18–50) | Third trimester | 12 | 0 | HBIG+  Vaccinatio |
|  |  |  |  | Control group | 138 | 32 (29–36) | 2.3 (1.6–3.1) (log10IU/mL) | 17 (12–24) | Control group | 73 | 1 | HBIG+  Vaccinatio |
| 13 | Pan, et al  (2016) | China  (Asians) | RCT | Tenofovir | 97 | 27.4±3.0 | 8.2±0.5 (log10IU/mL) | 23.0±22.4 | Gestation 30 to 32 weeks | 92 | 0 | HBIG+  Vaccinatio |
|  |  |  |  | Control group | 100 | 26.8±3.0 | 8.0±0.7 (log10IU/mL) | 20.5±15.4 | Control group | 88 | 6 | HBIG+  Vaccinatio |
| 14 | Liu, et al  (2016) | China  (Asians) | Prospective cohort study | Telbivudine | 50 | 27.88 ± 3.73 | 7.67 ± 0.79 (log10IU/mL) | 46.64 ± 58.74 | Before third trimester | 50 | 0 | HBIG+  Vaccinatio |
|  |  |  |  | Telbivudine | 32 | 28.31 ± 3.81 | 7.46 ± 0.73 (log10IU/mL) | 28.91 ± 38.48 | Third trimester | 32 | 1 | HBIG+  Vaccinatio |
|  |  |  |  | Control group | 78 | 27.46 ± 3.47 | 7.56 ± 0.57 (log10IU/mL) | 30.87 ± 28.99 | Control group | 78 | 19 | HBIG+  Vaccinatio |
| 15 | Sun Weihui(2015) | China | Cohort study | Ldt | 42 | 28．9士11．8 | 7.803±0.326 log10cps/ml | 138.8+62.6 | Gestation 12 weeks | 43 | 0 | HBIG+  Vaccinatio |
|  |  |  |  | Ldt | 41 | 29．7±9．8 | 7.916±0.165 log10cps/ml | 126.7±49.9 | Gestation 20-28weeks | 41 | 0 | HBIG+  Vaccinatio |
|  |  |  |  | Untreated | 45 | 27．5±12．9 | 7.852±0.245 log10cps/ml | 120.7±42.3 | Control group | 46 | 8 | HBIG+  Vaccinatio |
| 16 | G.-R. Han(2015) | China | Cohort study | Ldt | 257 | 27 (20–35) | 7.91 (6–9.0)log10 copies/mL | 21.45 (7.6–407.0) | Gestation Weeks 20–27 | 246 | 0 | HBIG+  Vaccinatio |
|  |  |  |  | Ldt | 105 | 28 (20–38) | 7.83 (6–9.1)log10 copies/mL | 17.1 (5.2–513.5) | Gestation Weeks 28–32 | 96 | 0 | HBIG+  Vaccinatio |
| 17 | Huey-Ling Chen(2015) | China，Taiwan | Cohort study | TDF | 62 | 32.41±3.12 | 8.25±0.45  (log10IU/mL) | 23.27±36.2 | Gestation 30-32weeks | 65 | 2 | HBIG+  Vaccinatio |
|  |  |  |  | Untreated | 56 | 32.45±3.2 | 8.24±0.35  (log10IU/mL) | 16.59±14.43 | Control group | 56 | 6 | HBIG+  Vaccinatio |
| 18 | Cheng Chuanying(2015) | China | Cohort study | Ldt | 42 | 29.7±8.9 | 7.7±0.3 log10 copies/mL | 276.6±106.3 | Gestation 12wks | 42 | 0 | HBIG+  Vaccinatio |
|  |  |  |  | Untreated | 40 | 27.5±6.6 | 7.8±0.5 log10 copies/mL | 252.9±91.7 | Control group | 40 | 7 |  |
| 19 | Zhou Yuejin(2015) | China | Cohort study | Ldt | 70 | 22±4 | >1×10^7^ copies/mL | ALTl00~300 U/L | Early pregnancy | 53 | 1 | HBIG+  Vaccinatio |
|  |  |  |  | Untreated | 45 | 21±4 | >1×10^7^ copies/mL | ALTl00~300 U/L | Control group | 34 | 6 | HBIG+  Vaccinatio |
| 20 | Hua Zhang(2014) | China | Cohort study | LDT | 252 | 29.56±4.78 | 7.69±00.44 log10 copies/mL | 34.85±27.17 | Gestation 28-30w | 257 | 0 | HBIG+  Vaccinatio |
|  |  |  |  | LAM | 51 | 29.56±4.78 | 7.62±0.37 log10 copies/mL | 34.85±27.17 | Gestation 28-30w | 52 | 0 | HBIG+  Vaccinatio |
|  |  |  |  | Untreated | 345 | 28.97±4.59 | 7.58±0.45 log10 copies/mL | 29.53±20.72 | Control group | 352 | 10 | HBIG+  Vaccinatio |
| 21 | Astrid-Jane Greenup1(2014) | Australia(Asian) | Cohort study | LAM | 62 | 30±8.5； | 7.94±0.78 log10IU/mL | TDF:28(22-36);Lam;22(18-30) | Gestation 32wks | 43 | 0 | HBIG+  Vaccinatio |
|  |  |  |  | TDF | 48 | 30±8.5； | 7.72±0.61 log10IU/mL | TDF:28(22-36);Lam;22(18-30) | Gestation 32wks | 44 | 1 | HBIG+  Vaccinatio |
|  |  |  |  | Untreated | 20 | 28±5 | 8.0±0.04 log10IU/mL | 25(17-31) | Control group | 10 | 2 | HBIG+  Vaccinatio |
| 22 | A. Ayres(2013) | Australia | Cohort study | LAM | 18 | NA | >10*7 IU/mL | NA | Gestation 32weeks | 18 | 0 | HBIG+  Vaccinatio |
|  |  |  |  | Untreated | 3 | NA | >10*7 IU/mL | NA | Control group | 3 | 1 | HBIG+  Vaccinatio |
| 23 | Mustafa Kemal Celen(2013) | Turkey | Cohort study | TDF | 21 | 28.2 ± 4.1 | 8.31 (log10IU/mL) | 52 (19-77) | Gestation 18～27w | 21 | 0 | HBIG+  Vaccinatio |
|  |  |  |  | Untreated | 24 | 26.9 ± 2.9 | 8.28 (log10IU/mL) | 56(22-71） | Control group | 23 | 2 | HBIG+  Vaccinatio |
| 24 | Zhang Liju(2009) | China | Cohort study | Ldt | 31 | NA | 7.38±0.81 log10cps/ml | NA | Gestation 28-32wk | 31 | 0 | HBIG+  Vaccinatio |
|  |  |  |  | Untreated | 30 | NA | 7.46±0.45 log10cps/ml | NA | Control group | 30 | 0 | HBIG+  Vaccinatio |
| 25 | W.-M. Xu(2009) | China | RCT | LAM | 89 | 26 (19–32) | DNA > 1000 MEq/mL | 0.4(0.1-5.0)ULN | Gestation 32wks | 56 | 10 | HBIG+  Vaccinatio |
|  |  |  |  | Untreated | 61 | 25 (20–36) | DNA > 1000 MEq/mL | 0.4(0.1-6)ULN | Control group | 59 | 23 | HBIG+  Vaccinatio |
| 26 | Yang Song(2008) | China | Cohort study | LAM 100mg | 20 | medain：27 | 3.6±2.5 log10cps/ml | ＜40 | Gestation 28wks | 19 | 2 | HBIG+  Vaccinatio |
|  |  |  |  | Untreated | 20 | medain：27 | 2.9±2.0 log10cps/ml | ＜40 | Control group | 20 | 2 | HBIG+  Vaccinatio |
| 27 | M. van Zonneveld(2003) | The Netherlands | Cohort study | LAM | 8 | 20(17-25) | ≥1.2 ×10^9^ geq/mL | normal | Gestation P34w | 8 | 1 | HBIG+  Vaccinatio |
|  |  | Mediteranean origin |  | Untreated | 24 | 23(16-34） | ≥1.2 ×10^9^ geq/mL | normal | Control group | 24 | 7 | HBIG+  Vaccinatio |
| 28 | Xiao-Mao Li(2003) | China | Cohort study | LAM | 43 | NA | 7.49±0.54 cps/ml Log10 HBV DNA | normal | Gestation 28w | 43 | 1 | HBIG+  Vaccinatio |
|  |  |  |  | Cntrol | 108 | NA | 7.05±1.29 cps/ml Log10 HBV DNA | normal | Control group | 108 | 18 | HBIG+  Vaccinatio |
| 29 | Zhi-Xian Chen(2017) | China | Prospective study | LDT | 23 | 28.1±6.7 | 7.2±0.7 log10 copies/mL | 89.3±104.2 | Second trimester | 23 | 0 | HBIG+  Vaccinatio |
|  |  |  |  | LDT | 18 | 28.1±6.7 | 7.2±0.7 log10 copies/mL |  | Third trimester | 18 | 1 | HBIG+  Vaccinatio |
|  |  |  |  | Untreated | 89 | 26.2±4.5 | 7.2±0.6 log10 copies/mL | 85±86.3 | Control group | 89 | 17 | HBIG+  Vaccinatio |
| 30 | Meilong Shen(2017) | China | Cohort study | LDT | 60 | NA | >10^5^ copies/mL | NA | Gestation 26week | 61 | 0 | HBIG+  Vaccinatio |
|  |  |  |  | LAM | 60 | NA | >10^5^ copies/mL | NA | Gestation 26week | 60 | 0 | HBIG+  Vaccinatio |
|  |  |  |  | Untreated | 28 | NA | >10^5^ copies/mL | NA | Untreated | 28 | 11 | HBIG+  Vaccinatio |
| 31 | Baoan Peng(2012) | China | Cohort study | LDT | 40 | NA | (5. 43 ± 0. 92) ×10^6^ copies/mL | Normal | Gestation 28week | 40 | 1 | HBIG+  Vaccinatio |
|  |  |  |  | Untreated | 40 | NA | (6. 75 ± 0. 77) ×10^6^ copies/mL | Normal | Untreated | 40 | 10 | HBIG+  Vaccinatio |
| 32 | Hanan M. Foaud(2019) | Egypt | Prospective observation study | LAM | 25 | 27.7 ± 4 | >10^5^ IU/ml | NA | Last Trimester | 9 | 0 | HBIG+  Vaccinatio |
|  |  |  |  | LAM | 9 | 27 ± 2.9 | >10^5^ IU/ml | NA | All through Pregnancy | 25 | 0 | HBIG+  Vaccinatio |
|  |  |  |  | Untreated | 39 | 27.4 ± 4.6 | NA | NA | Untreated | 39 | 1 | HBIG+  Vaccinatio |
| 33 | Jinfeng Liu(2019) | China | A prospective multicenter cohort study | LDT | 396 | 27.78± 3.56 | 7.89±0.66 Log10IU/mL | 45.79±66.34 | Gestation 24~28 week | 395 | 1 | HBIG+  Vaccinatio |
|  |  |  |  | TDF | 325 | 28.35 ± 4.35 | 7.68±0.70 Log10IU/mL | 53.34±71.87 | Gestation 24~28 week | 323 | 0 | HBIG+  Vaccinatio |
|  |  |  |  | Untreated | 136 | 27.14 ±4.72 | 7.71±0.79 Log10IU/mL | 41.16±62.46 | Untreated | 136 | 16 | HBIG+  Vaccinatio |
| 34 | Jianyong Zeng(2019) | Japan | Cohort study | LDT | 58 | 27.2±10.8 | ≥1.0×10^7^ copies/mL | 127.3±72.2 | Gestation 20~28 week | 58 | 0 | HBIG+  Vaccinatio |
|  |  |  |  | TDF | 51 | 26.5±9.5 | ≥1.0×10^7^ copies/mL | 143.3±104.6 | Gestation 20~28 week | 51 | 0 | HBIG+  Vaccinatio |
|  |  |  |  | Untreated | 36 | 25.7±10.9 | ≥1.0×10^7^ copies/mL | 132.3±78.3 | Untreated | 36 | 4 | HBIG+  Vaccinatio |
| 35 | Baofang Zhang(2019) | China | Retrospective study | LDT | 36 | NA | 4.84±2.01 Log10 IU/mL | NA | Gestation 24~28 week | 36 | 0 | HBIG+  Vaccinatio |
|  |  |  |  | TDF | 39 | NA | 5.08±1.99 Log10 IU/mL | NA | Gestation 24~28 week | 39 | 0 | HBIG+  Vaccinatio |
|  |  |  |  | Untreated | 75 | NA | 6.03±1.67 Log10 IU/mL | NA | Untreated | 75 | 75 | HBIG+  Vaccinatio |

**Table S2.** Risk of bias assessment using Cochrane Collaboration’s tool in the randomized controlled trial included in the meta-analysis.

| Study (year) | G.Jourdain(2018) | Calvin Q. Pan(2016) | W.-M. Xu(2009) |
| --- | --- | --- | --- |
| Random sequence generation (selection bias) | Low risk | Low risk | Low risk |
| Allocation concealment (selection bias) | Low risk | Low risk | Unclear |
| Blinding of participants and personnel (performance bias) | Low risk | Low risk | Low risk |
| Blinding of outcome assessment (detection bias) | Low risk | Low risk | Low risk |
| Incomplete outcome data addressed (attrition bias) | Unlear | Low risk | Unclear |
| Selective reporting (reporting bias) | Low risk | Low risk | Low risk |
| Anything else, ideally prespecified (other bias) | Unclear | Unclear | Unclear |

**Table S3.** Risk of bias measured by the Newcastle-Ottawa scale for the observational studies included in the meta-analysis.

| Study (year) | Representativeness of the exposed cohort | Selection of the non-exposed cohort | Ascertainment of exposure | Demonstration that outcome of interest was not present at baseline | Comparability of cohorts on the basis of the design or analysis | Assessment of outcomes | Was follow-up long enough for outcomes occur | Adequacy of follow up of cohorts | Total number of stars (Risk of bias) |
| --- | --- | --- | --- | --- | --- | --- | --- | --- | --- |
| Tianye He (2018) | No description | Drawn from the same community  as the exposed cohort | Record linkage | Yes | Study controls for any additional factors | Record linkage | Yes | Adequate | 7 |
| Cynthuja (2017) | Somewhat representative  of the community or population | Drawn from the same community  as the exposed cohort | Record linkage | Yes | Study controls for any additional factors | No description | Unclear | No description | 6 |
| Qiuju Sheng (2018) | No description | Drawn from the same community | Record linkage | Yes | Study controls for any additional factors | Record linkage | Yes | Adequate | 7 |
| Qiuju Sheng (2018) | Somewhat representative  of the community or population | as the exposed cohort | Record linkage | Yes | Study controls for any additional factors | Record linkage | Yes | Adequate | 8 |
| Yayun Lin (2018) | No description | Drawn from the same community | Record linkage | Yes | Study controls for any additional factors | Record linkage | Yes | Adequate | 7 |
| Y. Hu (2017) | Somewhat representative  of the community or population | as the exposed cohort | Record linkage | Yes | Study controls for any additional factors | Record linkage | Yes | No description | 7 |
| W.Yi (2017) | No description | Drawn from the same community | Record linkage | Yes | Study controls for any additional factors | Record linkage | Yes | Adequate | 7 |
| Weihui Sun(2017) | No description | as the exposed cohort | Record linkage | Yes | Study controls for any additional factors | Record linkage | Yes | Adequate | 7 |
| CQ Pan (2016) | No description | Drawn from the same community | Record linkage | Yes | Study controls for any additional factors | Record linkage | Yes | Adequate | 7 |
| Zhangmin Tan(2016) | No description | Drawn from the same community | Record linkage | Yes | Study controls for any additional factors | Record linkage | Yes | Adequate | 7 |
| G Samadik (2015) | No description | as the exposed cohort | Record linkage | Yes | Study controls for any additional factors | Record linkage | Yes | Adequate | 7 |
| Yingxia Liu (2016) | No description | Drawn from the same community | Record linkage | Yes | Study controls for any additional factors | Record linkage | Yes | Adequate | 7 |
| Weihui Sun(2015) | No description | as the exposed cohort | Record linkage | Yes | Study controls for any additional factors | Record linkage | Yes | Adequate | 7 |
| GR. Han (2015) | No description | Drawn from the same community | Record linkage | Yes | Study controls for any additional factors | Record linkage | Yes | Adequate | 7 |
| Huey-Ling Chen(2015) | Somewhat representative  of the community or population | as the exposed cohort | Record linkage | Yes | Study controls for any additional factors | Record linkage | Yes | Adequate | 8 |
| Chuanying Chen (2015) | No description | Drawn from the same community | Record linkage | Yes | Study controls for any additional factors | Record linkage | Yes | Adequate | 7 |
| Yuejin Zhou(2015) | No description | as the exposed cohort | Record linkage | Yes | Study controls for any additional factors | Record linkage | Yes | Adequate | 6 |
| Hua Zhang(2014) | No description | Drawn from the same community | Record linkage | Yes | Study controls for any additional factors | Record linkage | Yes | Adequate | 7 |
| AJ Greenup (2014) | Somewhat representative  of the community or population | as the exposed cohort | Record linkage | Yes | Study controls for any additional factors | Record linkage | Yes | No description | 7 |
| A.Ayres (2014) | No description | Drawn from the same community | Record linkage | Yes | Study controls for any additional factors | Record linkage | Yes | Adequate | 7 |
| M Yu(2011) | No description | as the exposed cohort | Record linkage | Yes | Study controls for any additional factors | Record linkage | Yes | Adequate | 7 |
| Liju Zhang (2009) | No description | Drawn from the same community | Record linkage | Yes | Study controls for any additional factors | Record linkage | Yes | Adequate | 7 |
| Song Yang(2008) | No description | as the exposed cohort | Record linkage | Yes | Study controls for any additional factors | Record linkage | Yes | Adequate | 7 |
| M Vanv Zonneveld(2003) | No description | Drawn from the same community | Record linkage | Yes | No description | Record linkage | Yes | Adequate | 6 |
| Xiao Mao Li(2003) | No description | as the exposed cohort | Record linkage | Yes | Study controls for any additional factors | Record linkage | No description | Adequate | 6 |
| Zhi-Xian Chen(2017) | No description | as the exposed cohort | Record linkage | Yes | Study controls for any additional factors | Record linkage | Yes | Adequate | 7 |
| Meilong Shen(2017) | Somewhat representative  of the community or population | as the exposed cohort | Record linkage | Yes | Study controls for any additional factors | Record linkage | Yes | Unclear | 6 |
| Baoan Peng(2012) | Somewhat representative  of the community or population | as the exposed cohort | Record linkage | Yes | Study controls for any additional factors | Record linkage | Yes | Adequate | 7 |
| Hanan M. Foaud(2019) | Somewhat representative  of the community or population | as the exposed cohort | Record linkage | Record linkage | Study controls for any additional factors | Record linkage | Yes | Adequate | 8 |
| Jinfeng Liu(2019) | Somewhat representative  of the community or population | as the exposed cohort | Record linkage | Record linkage | Study controls for most important factor | Record linkage | Yes | Adequate | 8 |
| Jianyong Zeng(2019) | Somewhat representative  of the community or population | as the exposed cohort | Record linkage | Record linkage | Study controls for most important factor | Record linkage | Yes | Adequate | 8 |
| Baofang Zhang(2019) | Somewhat representative  of the community or population | as the exposed cohort | Record linkage | Yes | No description | Record linkage | Yes | Unclear | 6 |

**Table S4.** Quality of evidence summary for non-randomized controlled trails.

| Intervention | Outcomes | No.of participants | Quality of the evidence(GRADE) | | |
| --- | --- | --- | --- | --- | --- |
|  |  |  | Direct | Indirect | Network |
| Infant efficacy outcomes | | | | | |
| Antiviral~28week vs 28-32week | MTCT | 6738 | ⨁ ◯◯◯  VERY LOW | ⨁⨁◯◯  LOW | ⨁⨁◯◯  LOW |
| Infant safety outcomes | | | | | |
| Antiviral~28week vs 28-32week | Apgar score(1 minute)<8 | 2208 | ⨁ ◯◯◯  VERY LOW | ⨁⨁◯◯  LOW | ⨁⨁◯◯  LOW |
|  | Fetal Death | 249 | NA | ⨁ ◯◯◯  VERY LOW | ⨁ ◯◯◯  VERY LOW |
|  | Congenital Malformation | 4295 | ⨁ ◯◯◯  VERY LOW | ⨁⨁◯◯  VERY LOW | ⨁⨁◯◯  VERY LOW |
|  | Low Birth Weight | 1687 | ⨁ ◯◯◯  VERY LOW | ⨁ ◯◯◯  VERY LOW | ⨁ ◯◯◯  VERY LOW |
|  | Prematurity Rate | 3348 | ⨁ ◯◯◯  VERY LOW | ⨁ ◯◯◯  VERY LOW | ⨁ ◯◯◯  VERY LOW |
| Maternal Efficacy Outcomes | | | | | |
| Antiviral~28week vs 28-32week | HBV DNA Suppression at Delivery | 3067 | ⨁ ⨁ ◯◯ LOW | ⨁⨁◯◯  LOW | ⨁⨁◯◯  LOW |
|  | ALT Normaliztion at Deilivery | 1985 | ⨁⨁◯◯  LOW | ⨁⨁◯◯  LOW | ⨁⨁◯◯  LOW |
|  | HBeAg Seroconversion | 679 | ⨁ ◯◯◯  VERY LOW | ⨁⨁◯◯  LOW | ⨁⨁◯◯  LOW |
| Maternal safety Outcomes | | | | | |
| Antiviral~28week vs 28-32week | Cesarean Section Rate | 5123 | ⨁ ◯◯◯  VERY LOW | ⨁ ◯◯◯  VERY LOW | ⨁ ◯◯◯  VERY LOW |
|  | Postpartum hemorrhage rate | 3961 | ⨁ ◯◯◯  VERY LOW | ⨁ ◯◯◯  VERY LOW | ⨁ ◯◯◯  VERY LOW |
|  | Threathened Abortion | 569 | NA | NA | NA |
|  | Elevated Creatine Kinase | 1317 | NA | ⨁ ◯◯◯  VERY LOW | ⨁ ◯◯◯  VERY LOW |
|  | Gestational hypertension | 793 | NA | NA | NA |
|  | Gestational diabetes mellitus | 926 | ⨁ ◯◯◯  VERY LOW | ⨁ ◯◯◯  VERY LOW | ⨁ ◯◯◯  VERY LOW |
|  | Membrane prerupture | 485 | NA | NA | NA |
|  | Oligohydramnios | 1139 | ⨁ ◯◯◯  VERY LOW | ⨁ ◯◯◯  VERY LOW | ⨁ ◯◯◯  VERY LOW |
|  | Polyhydramnios | 1139 | ⨁ ◯◯◯  VERY LOW | ⨁ ◯◯◯  VERY LOW | ⨁ ◯◯◯  VERY LOW |
|  | Meconium staining of the amniotic fluid (III degree) | 645 | ⨁ ◯◯◯  VERY LOW | ⨁ ◯◯◯  VERY LOW | ⨁ ◯◯◯  VERY LOW |

**Figure S1 Funnel plot of pair-wised meta-analysis of MTCT for non-RCTs, comparing different timing versus control group. A) ~28week versus control. B) 28~32week versus control.**

**A**

**
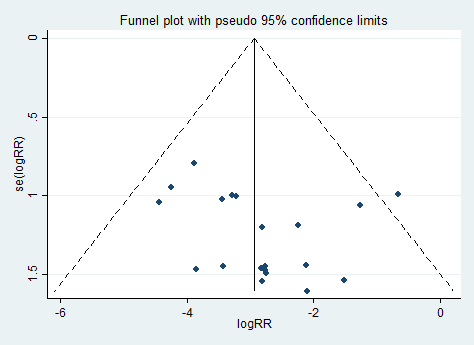
**

**B**

**
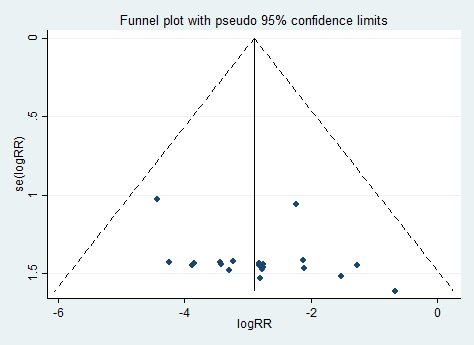
**

**Figure S2 Forest plot of direct meta-analysis of MTCT for non-RCTs, comparing different timing versus control group. A) ~14week versus control. B) 14~28week versus control.**

**A**

**
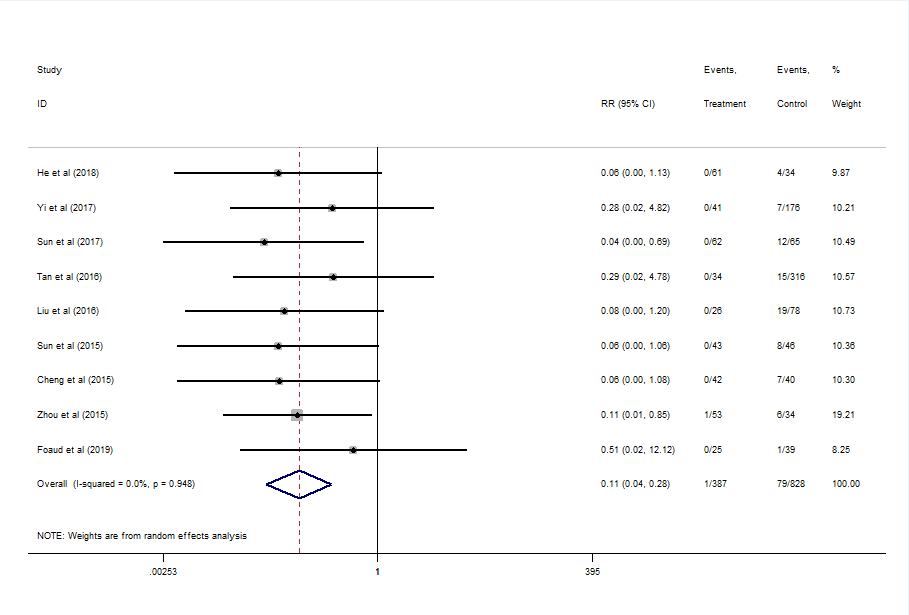
**

**B**

**
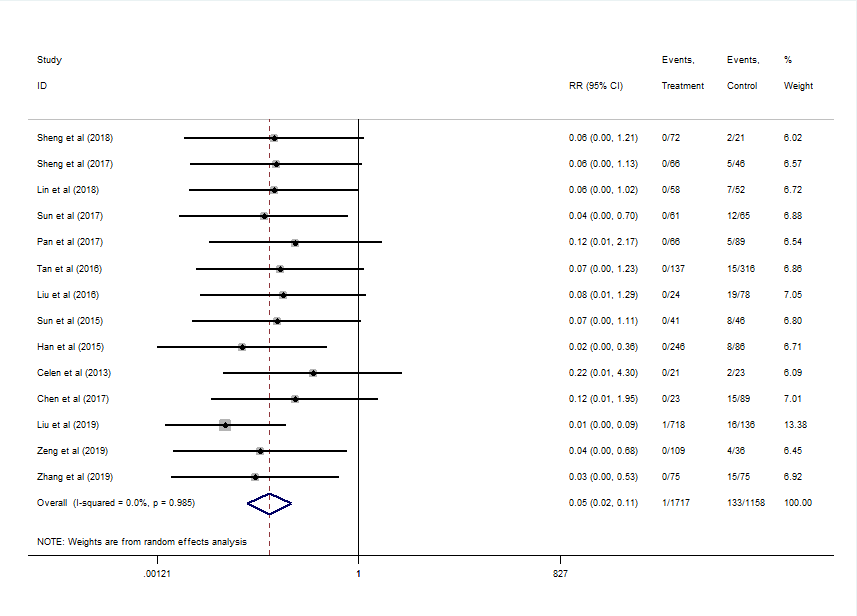
**

**Figure S3 Network plot of MTCT for different agents initiated in pregnancy.**

**
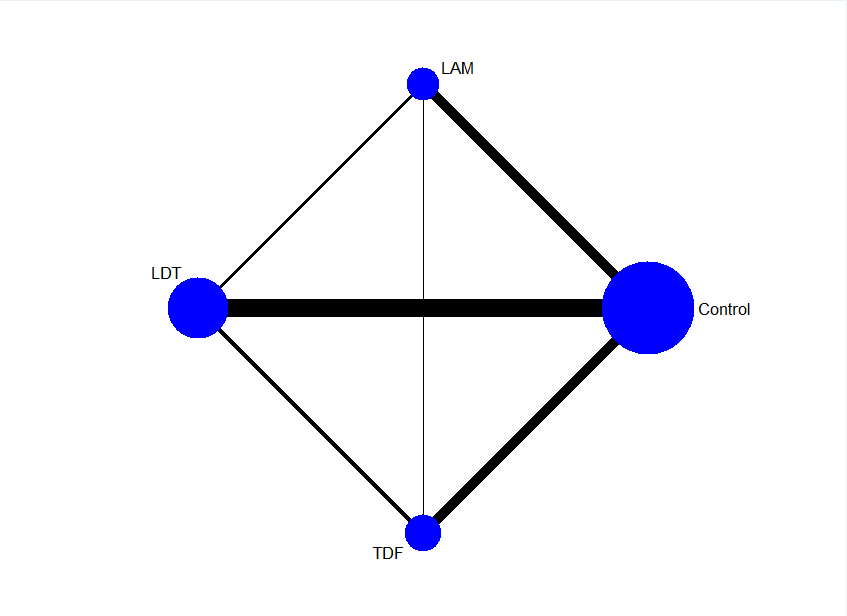
**

**Figure S4 A. Network plot of MTCT for different agents used A) before third trimester and B) used in third trimester on pooled analysis.**

**A**

**
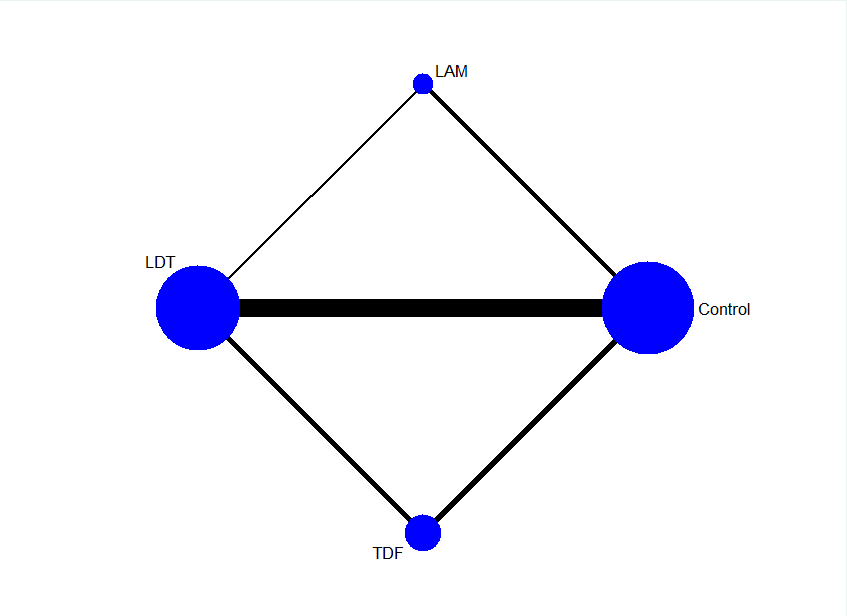
**

**B**

**
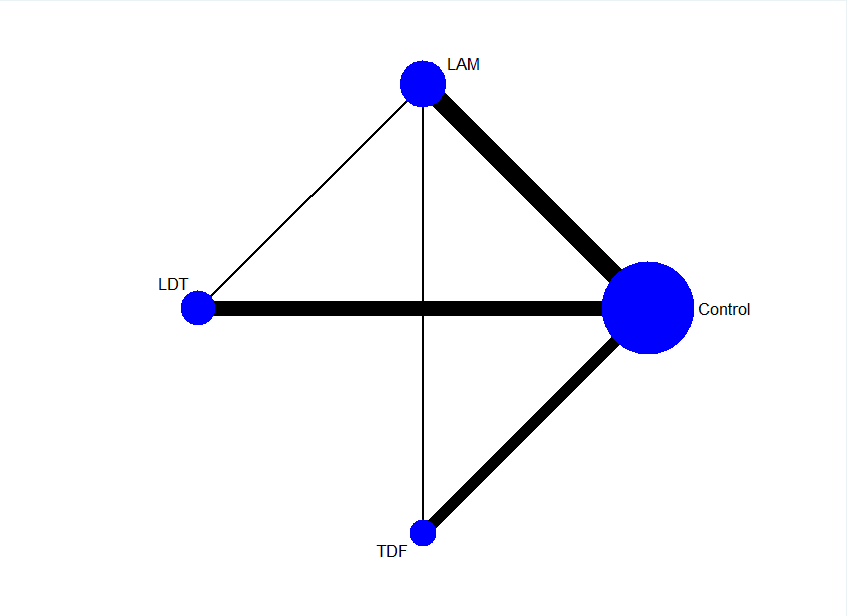
**

**Figure S5 Rank probabilities among antiviral agents for interrupting HBV MTCT based on the pooled network meta-analysis.**

**
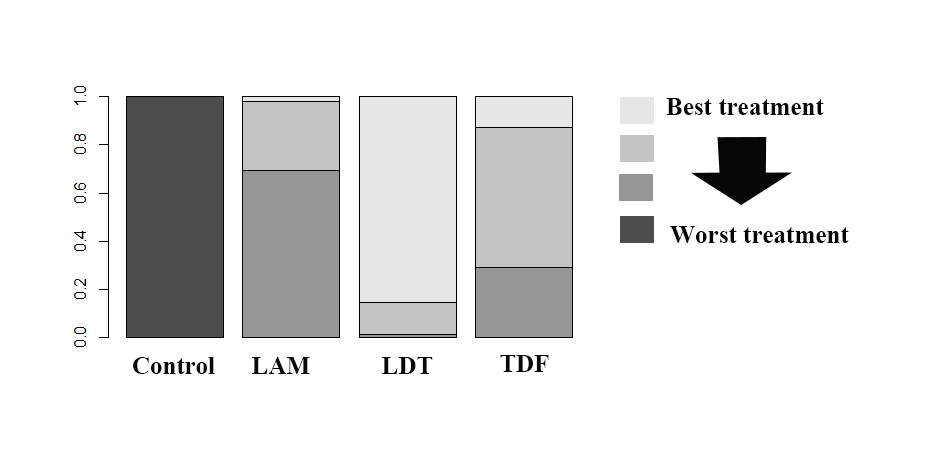
**

**Figure S6 Network plot of MTCT for any agents among different trimesters in non-RCTs.**

**
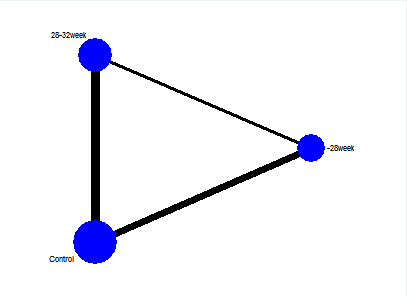
**

**Figure S7 Network plot for MTCT comparing any agents in first, second, third trimester versus control at 6-12 months after delivery in non-RCTs.**

**
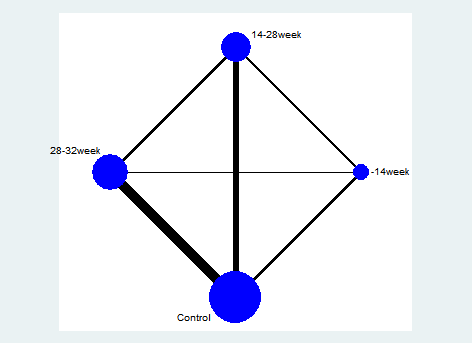
**

**Figure S8 Rank probabilities of the timing on starting antiviral agents for interrupting HBV MTCT based on the network meta-analysis in non-RCTs.**

**
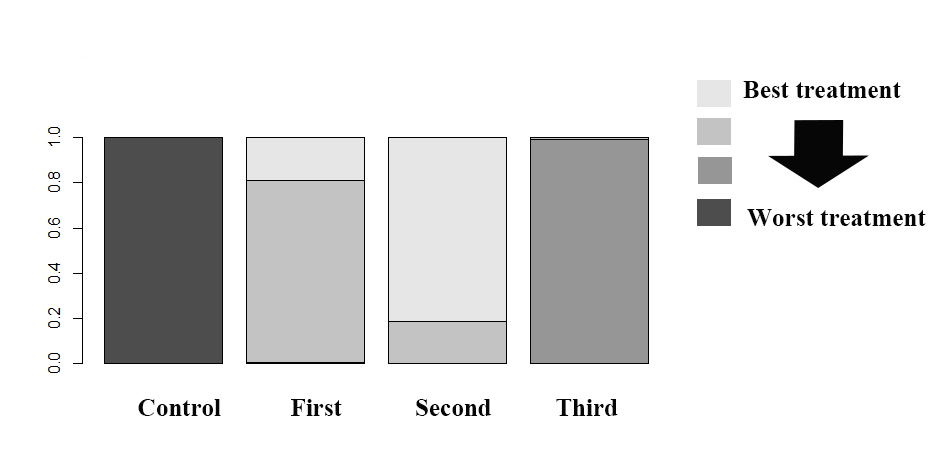
**

**Figure S9 Forest plot for network meta-analysis of MTCT of any agents applied for HBeAg positive mothers among different trimesters.**

**
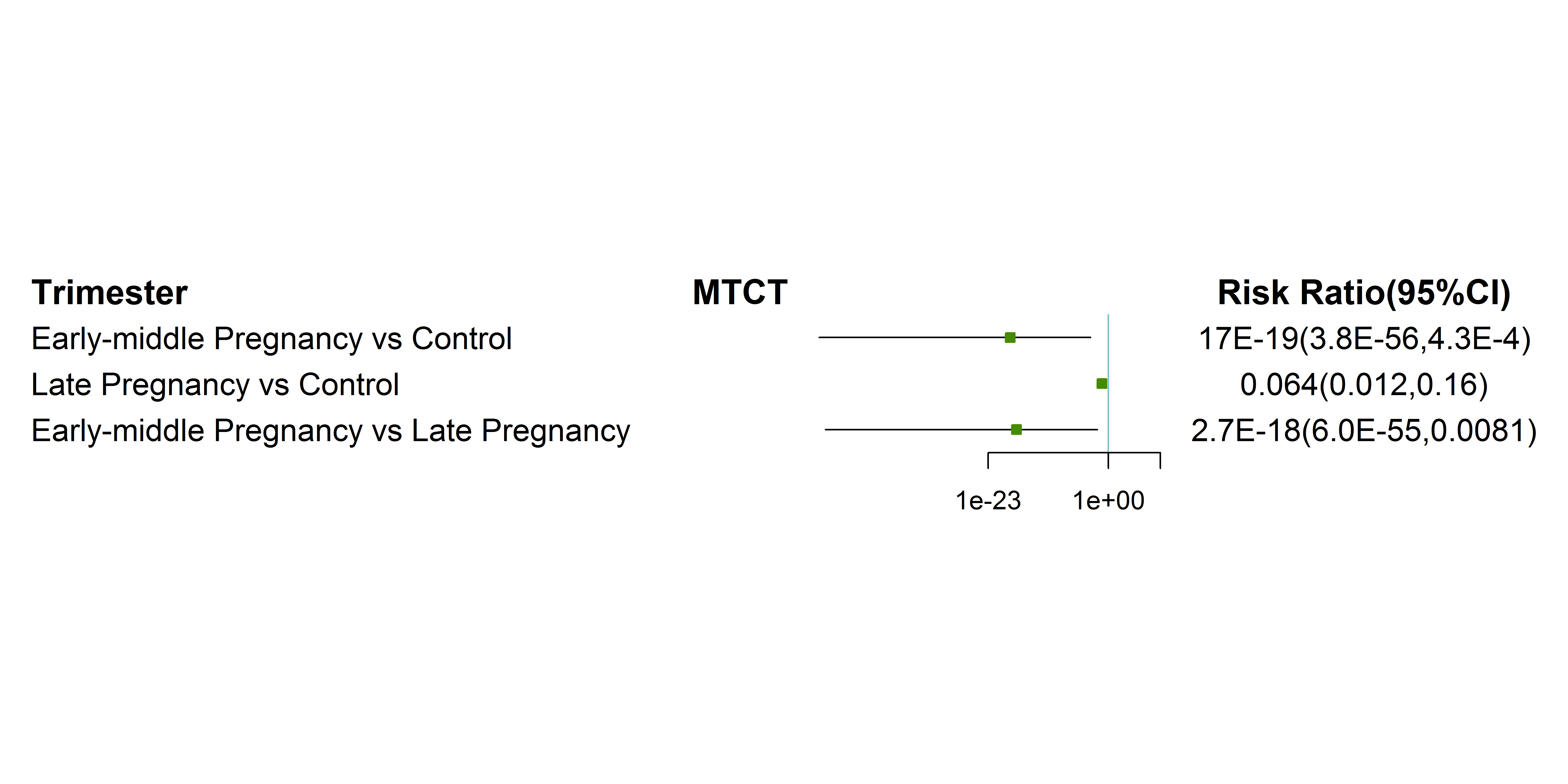
**

**Figure S10 Forest plots of maternal efficacy outcomes for non-RCTs comparing antiviral treatment in different trimesters versus control.**

**
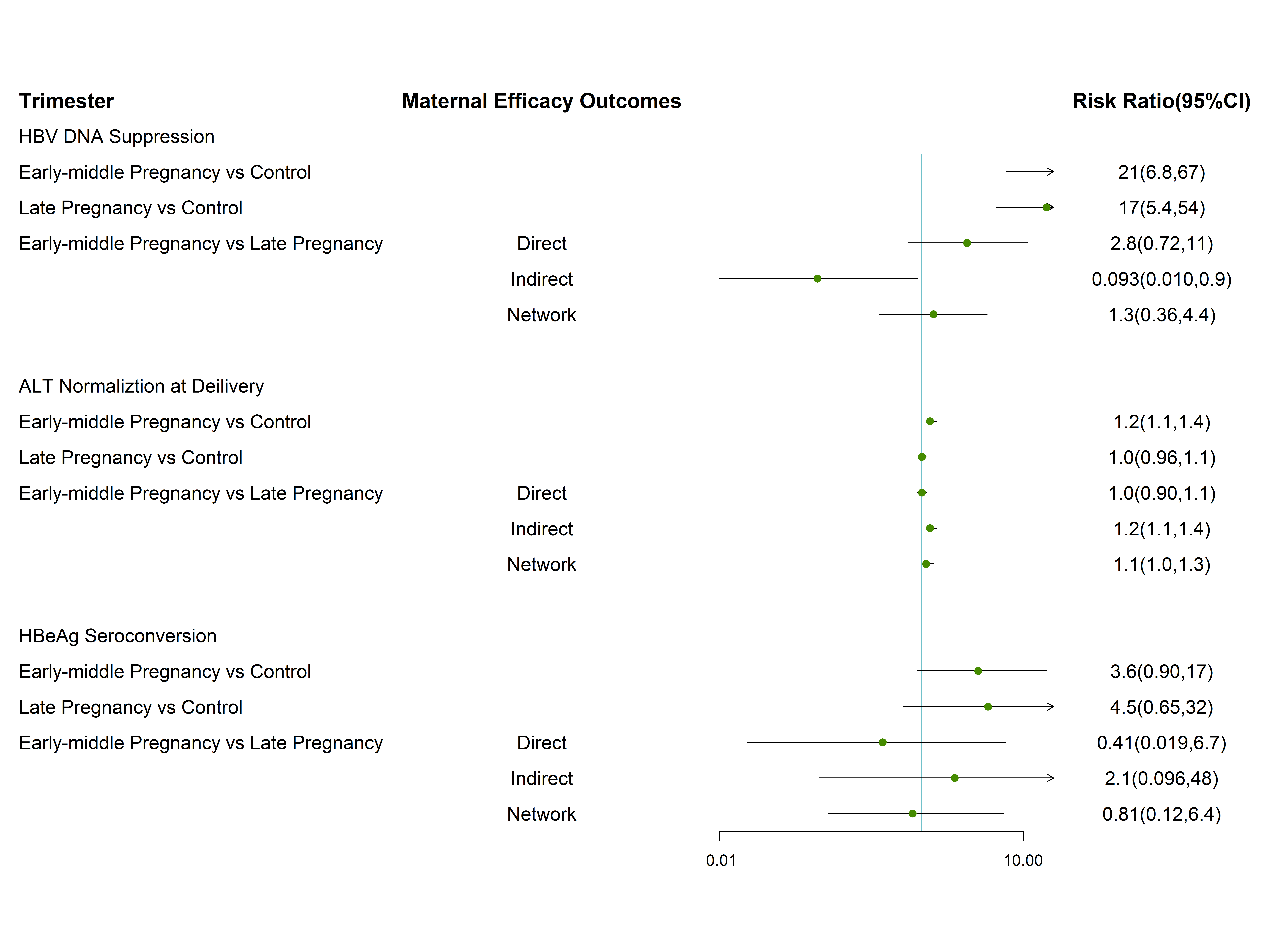
**

**Figure S11 Forest plots of infant safety outcomes for non-RCTs comparing antiviral treatment in different trimesters versus control.**

**

**

**Figure S12 Forest plots of maternal safety outcomes for non-RCTs comparing antiviral treatment in different trimesters versus control**


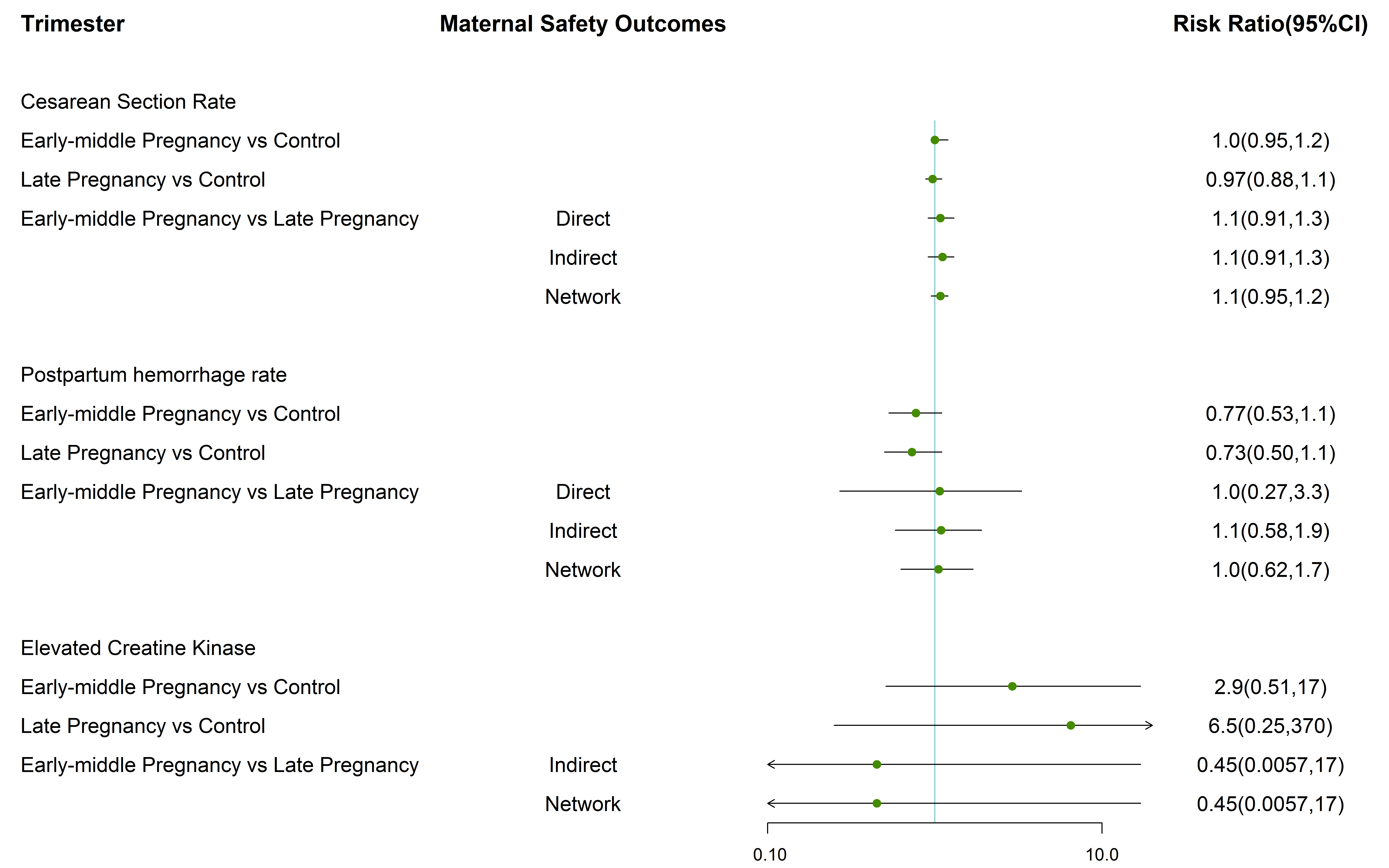


(Continue)


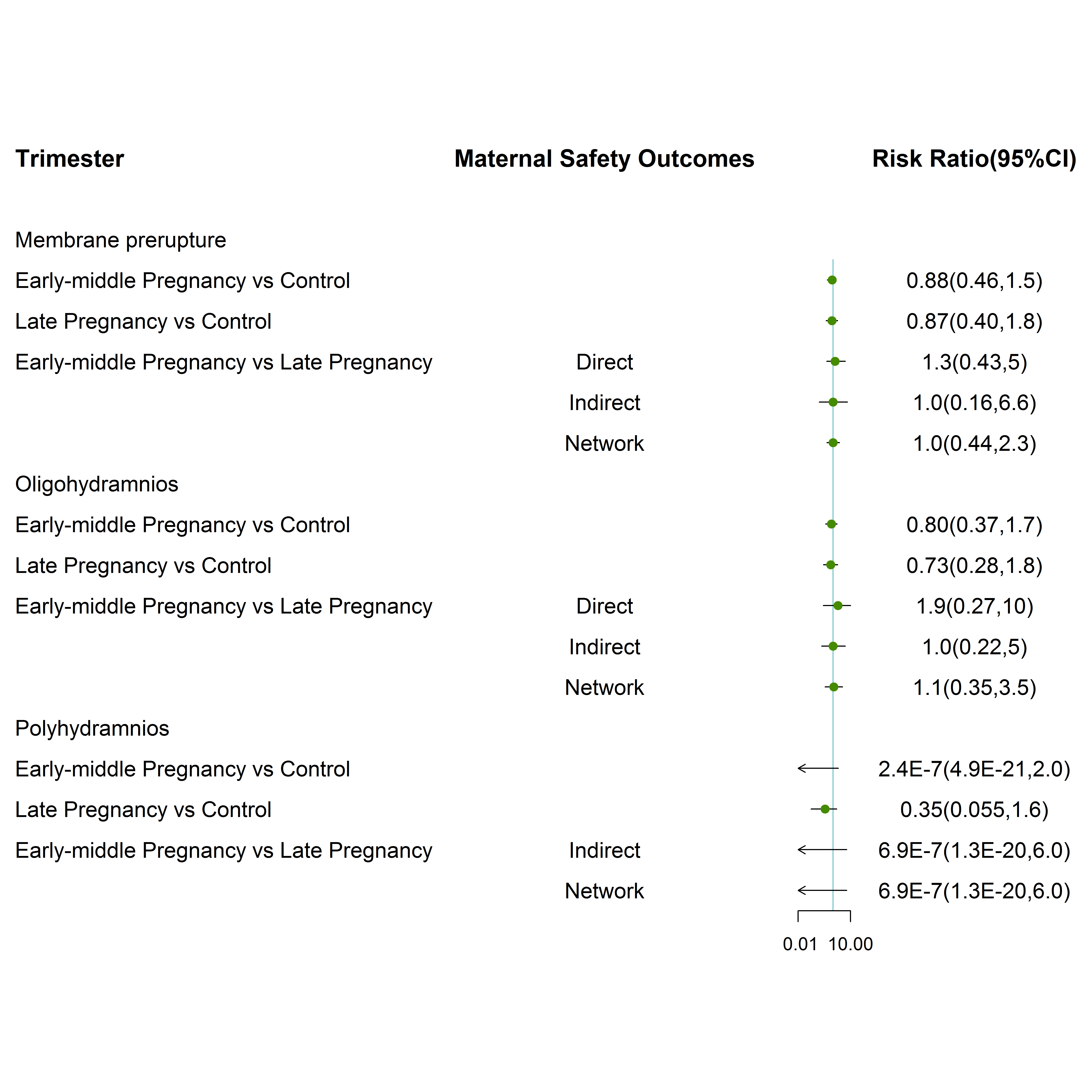


(Continue)


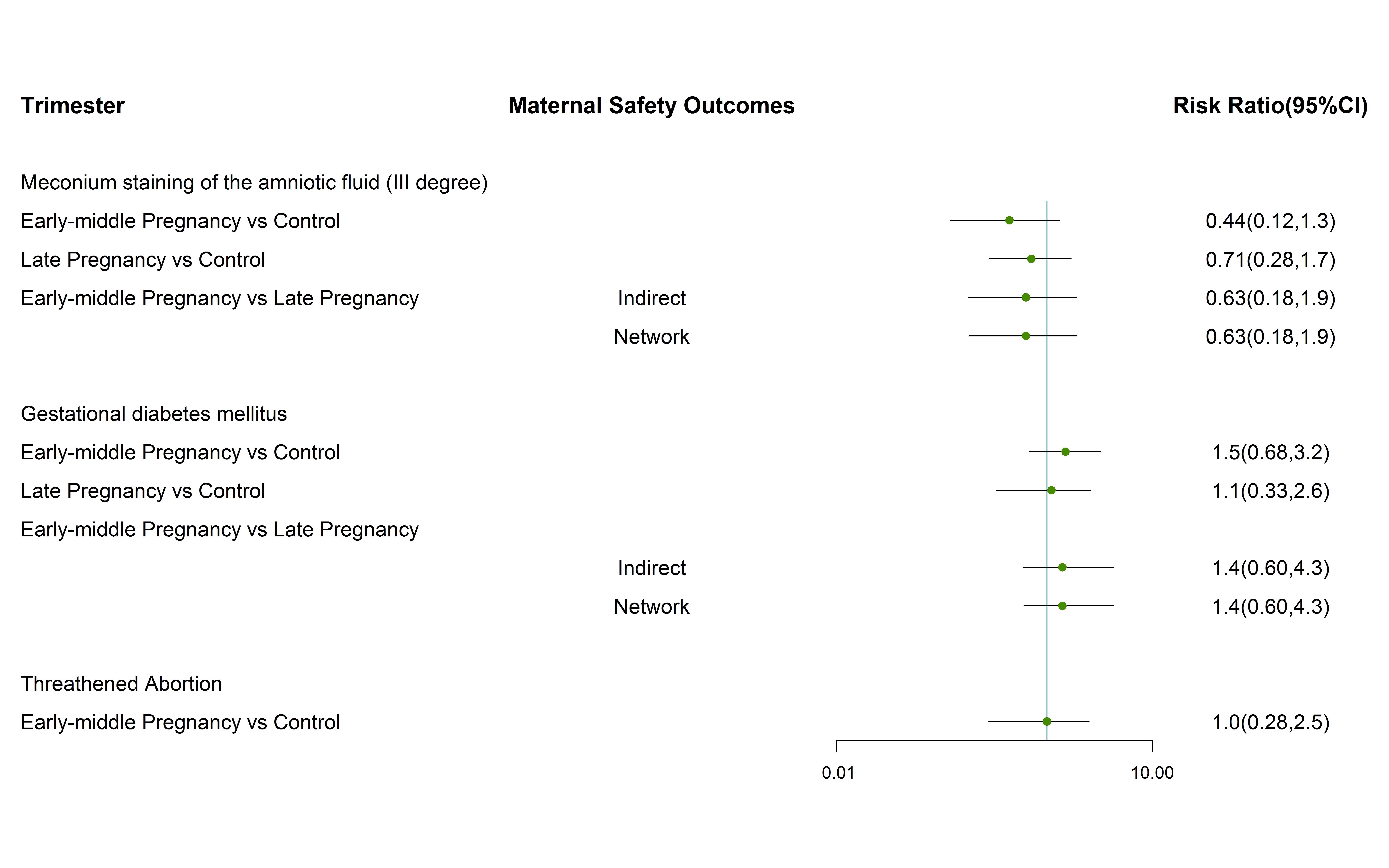


1 Hutton B, Salanti G, Caldwell DM, Chaimani A, Schmid CH, Cameron C*, et al.* The PRISMA extension statement for reporting of systematic reviews incorporating network meta-analyses of health care interventions: checklist and explanations. Annals of internal medicine 2015;**162**:777-84.

2 Higgins JP, Altman DG, Gotzsche PC, Juni P, Moher D, Oxman AD*, et al.* The Cochrane Collaboration's tool for assessing risk of bias in randomised trials. BMJ (Clinical research ed) 2011;**343**:d5928.

3 Stang A. Critical evaluation of the Newcastle-Ottawa scale for the assessment of the quality of nonrandomized studies in meta-analyses. European journal of epidemiology 2010;**25**:603-5.

4 Rucker G, Schwarzer G. Reduce dimension or reduce weights? Comparing two approaches to multi-arm studies in network meta-analysis. Statistics in medicine 2014;**33**:4353-69.

5 Higgins JP, Thompson SG, Deeks JJ, Altman DG. Measuring inconsistency in meta-analyses. BMJ (Clinical research ed) 2003;**327**:557-60.

6 Egger M, Davey Smith G, Schneider M, Minder C. Bias in meta-analysis detected by a simple, graphical test. BMJ (Clinical research ed) 1997;**315**:629-34.

7 Krahn U, Binder H, Konig J. A graphical tool for locating inconsistency in network meta-analyses. BMC medical research methodology 2013;**13**:35.

8 Puhan MA, Schunemann HJ, Murad MH, Li T, Brignardello-Petersen R, Singh JA*, et al.* A GRADE Working Group approach for rating the quality of treatment effect estimates from network meta-analysis. BMJ (Clinical research ed) 2014;**349**:g5630.
